# Optical and Plasmonic Properties of High-Electron-Density Epitaxial and Oxidative Controlled Titanium Nitride Thin Films

**DOI:** 10.1021/acs.jpcc.4c06969

**Published:** 2025-02-03

**Authors:** Ikenna Chris-Okoro, Sheilah Cherono, Wisdom Akande, Swapnil Nalawade, Mengxin Liu, Catalin Martin, Valentin Craciun, R. Soyoung Kim, Johannes Mahl, Tanja Cuk, Junko Yano, Ethan Crumlin, J. David Schall, Shyam Aravamudhan, Maria Diana Mihai, Jiongzhi Zheng, Lei Zhang, Geoffroy Hautier, Dhananjay Kumar

**Affiliations:** † Department of Mechanical Engineering, North Carolina Agricultural and Technical State University, Greensboro, North Carolina 27411, United States; ‡ Joint School of Nanoscience and Nanoengineering, North Carolina Agricultural and Technical State University, Greensboro, North Carolina 27401, United States; § School of Theoretical & Applied Sciences, Ramapo College of New Jersey, Mahwah, New Jersey 07430, United States; ∥ National Institute for Laser, Plasma, and Radiation Physics and Extreme Light Infrastructure for Nuclear Physics, Romania, 060042 Magurele, Romania; ⊥ Chemical Sciences Division, Lawrence Berkeley National Laboratory, Berkeley, California 94720, United States; # Department of Chemistry, University of Colorado, Boulders, Colorado 80309, United States; ¶ Horia Hulubei National Institute for Physics and Nuclear Engineering, Magurele, Ilfov 077125, Romania; ∇ Department of Physics, National University of Science and Technology Politehnica Bucharest, Bucharest, Romania 060042, Romania; ○ Thayer School of Engineering, Dartmouth College, Hanover, New Hampshire 03755, United States

## Abstract

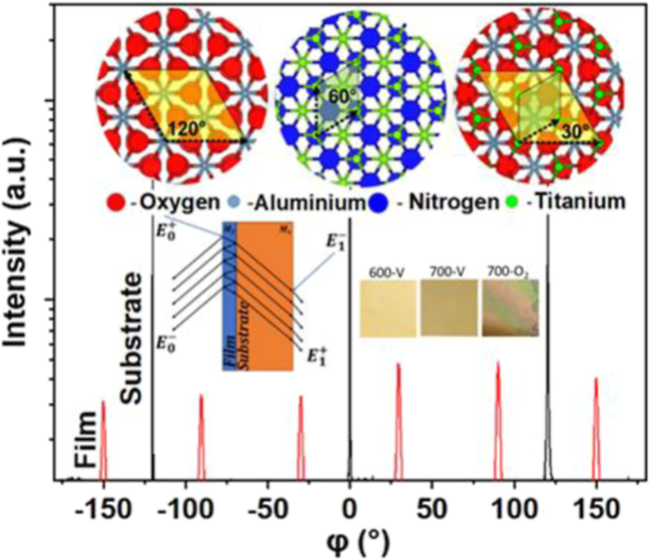

The present paper reports on the fabrication, detailed structural characterizations, and theoretical modeling of titanium nitride (TiN) and its isostructural oxide derivative, titanium oxynitride (TiNO) thin films that have excellent plasmonic properties and that also have the potential to overcome the limitation of noble metal and refractory metals. The TiNO films deposited at 700 °C in high vacuum conditions have the highest reflectance (*R* = ∼ 95%), largest negative dielectric constant (ε_1_ = −161), and maximal plasmonic figure of merit (FoM = −ε_1_/ε_2_) of 1.2, followed by the 600 °C samples deposited in a vacuum (*R* = ∼ 85%, ε_1_ = −145, FoM = 0.8) and 700 °C–5 mTorr sample (*R* = ∼ 82%, ε_1_ = −8, FoM = 0.3). To corroborate our experimental observations, we calculated the phonon dispersions and Raman active modes of TiNO by using the virtual crystal approximation. From the experimental and theoretical studies, a multilayer optical model has been proposed for the TiN/TiNO epitaxial thin films for obtaining individual complex dielectric functions from which many other optical parameters can be calculated. The advantages of oxide derivatives of TiN are the continuation of similar free electron density as in TiN and the acquisition of additional features such as oxygen-dependent semiconductivity with a tunable bandgap.

## 1 Introduction

Harnessing the time-independent interaction between light and matter in nonlinear optical processes is essential for applications such as the identification of molecules, photocatalysis, photovoltaics, and plasmonics.^1−4^ Several materials and material synthesis strategies have been proposed for these applications. While high-performance material development is important, it is also essential to enhance and understand their light–solid interactions^5−10^ for efficient overall energy utilization.^11,12^ The field of plasmonics has enabled extraordinary enhancement of electromagnetic radiation beyond the diffraction limit of light, a common trait of conventional optics. Though the coupling of light to electronic charge in certain metals has been investigated for decades, the advancement in surface-enhanced Raman spectroscopy in the last 25 years has improved our knowledge of surface plasmons, which has the ability to confine light to specific wavelengths.^13−15^ These plasmons are oscillations of the free electron gas system, often at optical frequencies but confined to the metal surface, rather than having collective oscillation of a gas.^16^ In recent years, better control of the surface plasmon resonance has been demonstrated by engineering the material structure, surface periodicity, and oxidation state.^16−18^ These controls have led to innovative plasmonic-based nanometer-scale devices with enhanced performance and terahertz operational bandwidths.^19−23^ It should be noted that besides materials, structure, periodicity, and oxidation states, the shape and architecture of materials also have a profound effect on the properties of these materials.^24−37^ For example, in nanowire shape, the permittivity (ε) is different along the axes parallel or perpendicular to the propagation of light, where ε_∥_ < 0, ε_⊥_>0. This relationship is reversed for thin-film materials, where ε_∥_ > 0, ε_⊥_ < 0. When signs of the two components of permittivity are opposite, the isofrequency contours are unbounded and result in regular hyperbola for 2D thin-film configuration as opposed to inverted hyperbolas in the case of 1D nanowires.^10^ Thus, by balancing the frequency-dependent permittivity of the dielectric (positive) and metallic materials (negative) together with the geometrical parameters of the real part of ε_∥_ and ε_⊥_, one can obtain ε_∥_ and ε_⊥_ of opposite signs in the same material.

**Figure 1:**
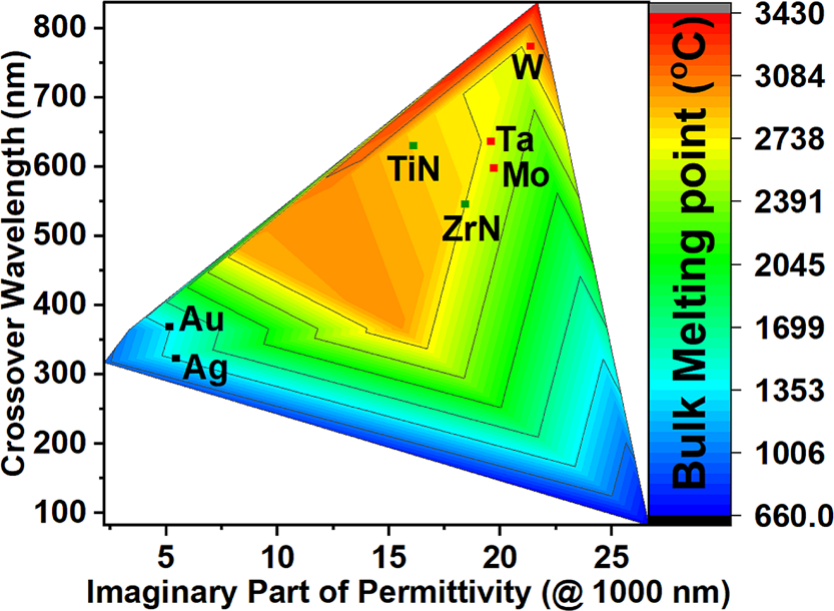
Comparison of selected transition metal nitrides (e.g., TiN, ZrN) with plasmonic noble metals (e.g., Au, Ag) and refractory (e.g., W, Ta, Mo) metals.^24,37^

Large free carrier density and oscillation of the free charge carriers in noble metals such as silver and gold provide the opportunity for convenient tuning of the plasmonic properties,^23,38−41^ and therefore, noble metals are still at the center of fundamental and applied plasmonic research. At the same time, the applications of noble metals in plasmonic devices are faced with several challenges, such as high cost and low mechanical and thermal stability, especially when working in harsh operational conditions.^21^ Refractory metals such as tantalum (Ta), molybdenum (Mo), and tungsten (W) have been used as alternative candidates in plasmonic applications.^37^ However, these metals lack plasmonic properties in the visible range and exhibit poor resonances in the near-infrared region.^37,39^ The present work reports on the structural and plasmonic properties of pulsed laser-deposited titanium nitride (TiN) and titanium oxynitride (TiNO) thin films.^42,43^ The selection of TiN stems from its relatively lower cost, higher free electron gas density (∼10^22^ cm^–3^), and conductivity (1.25 × 10^4^ Ω^–1^ cm^–1^)^44^ in comparison to conductive transition metal nitride (TMN) ceramics made up of group IVB-VB-VIB transition metals, such as chromium nitride (8.33 × 10^3^ Ω^–1^ cm^–1^), vanadium nitride (1.0 × 10^4^ Ω^–1^ cm^–1^), hafnium nitride (8.00 × 10^3^ Ω^–1^ cm^–1^), and zirconium nitride (6.25 × 10^3^ Ω^–1^ cm^–1^).^45−49^ The electron-free density in TiN is similar to that of Au or Ag (10^22^–10^23^ cm^–3^); TiN also embodies the characteristics of common refractory metals (Figure 1).^50,51^ The availability of free electrons in TiN is brought about by the electronic configurations of ions involved and the bonding between them.^52^ The other material advantage offered by TiN that enables new optoelectronic properties and device physics is its carrier concentration suitable for high plasma frequency (ω_p_ = (*Ne*^2^/ε_0_*m*_e_)^1/2^) and tunable bandgaps to reduce interband transition losses,^53^ where ω_p_ is the plasma frequency, *N* is the number of electrons, *e* is the electronic charge, ε_0_ is the permittivity of free space, and *m*_e_ is the mass of an electron.

These advantages of TiN are taken to the next level by transforming TiN to semiconducting titanium oxynitrides (TiNO) with precise control in oxygen composition that can, in turn, be used to tune the electronic band structure of TMSs, opening another new dimension to TMN-based plasmonics and metamaterials research. There is a sizable literature on the plasmonic properties of TMNs and even less literature on their oxynitrides, the interest in which continues to grow.^54^ However, most of the research in this direction is targeted at producing plasmonic materials that have quality factors of localized surface plasmon resonances as close as possible to those of gold, silver, and copper. Some studies have focused on the operational temperature effects across various thicknesses,^55^ thickness effect,^56−58^ doping effect,^59^ and effect of material geometries^60^ on the plasmonic characteristics. In this study, the effect of varying oxidation levels of TiN and crystalline quality on the plasmonic performance of TiN and TiNO thin films is presented, with theoretical calculations performed to understand how these elemental processing parameters affect the overall performance of transitional metal nitride and oxynitride thin films.

## 2 Experimental Methods

Titanium nitride and titanium oxynitride thin films were deposited on *c*-plane sapphire (0001) substrates by using a pulsed laser deposition (PLD) technique. The details of the PLD processing parameters are described in Supporting Information Note S1 and our earlier publications.^28−31^ The surface morphology of TiN and TiNO films was investigated by using atomic force microscopy (AFM) (Asylum Jupiter XR). The electrical resistivity was determined by using an Ossila T2001A standard four-probe measurement. The unit lattice models were simulated using Visualization for Electronic and Structural Analysis (VESTA). The film orientation, thickness, and crystallinity were analyzed using an X-ray diffractometer (Rigaku Smartlab XRD) with a high-flux Cu Kα X-ray source (λ = 0.154 nm). XPS measurements were carried out using a Thermo Fisher ESCALAB Xi + instrument working with Al Kα monochromatic radiation. Raman spectroscopy measurements were carried out using a WiTec alpha 300R Confocal Raman Microscope at a laser excitation wavelength of 532 nm (green visible light). High-resolution X-ray photoelectron spectroscopy (XPS) scans were recorded for Ti 2p, N 1s, C 1s, and O 1s core levels to accurately quantify the oxidized, partially oxidized, and unoxidized phases of TiN by taking care to avoid the common errors frequently encountered right from the data collection to subsequent analysis. A precise quantification of these phases is important in understanding the resulting properties of TiNO compounds formed at higher deposition temperatures and in the presence of ambient oxygen. The details of XPS fitting approaches^61^ are described in the Experimental Sections. The elemental composition of the TiN and TiNO films was also determined using non-Rutherford backscattering spectrometry (NRBS) with ^4^He^2+^ ions at 3.043 MeV and 3.7 MeV.^62−64^ The Supporting Information contains the details of NRBS analysis and data interpretation. Ab-initio calculations based on density functional theory (DFT) using the Vienna Ab-initio Simulation Package (VASP) were carried out as discussed in Supporting Information Note S2. To ensure the authenticity and reproducibility of the data presented in this study, samples were deposited twice under identical conditions, and then, their properties (thickness, crystallinity, four-probe resistivity, Raman Spectra, NRBS, and XPS compositions) were measured at least twice in different locations.

## 3 Results and Discussion

### 3.1 Structural Properties

**Figure 2:**
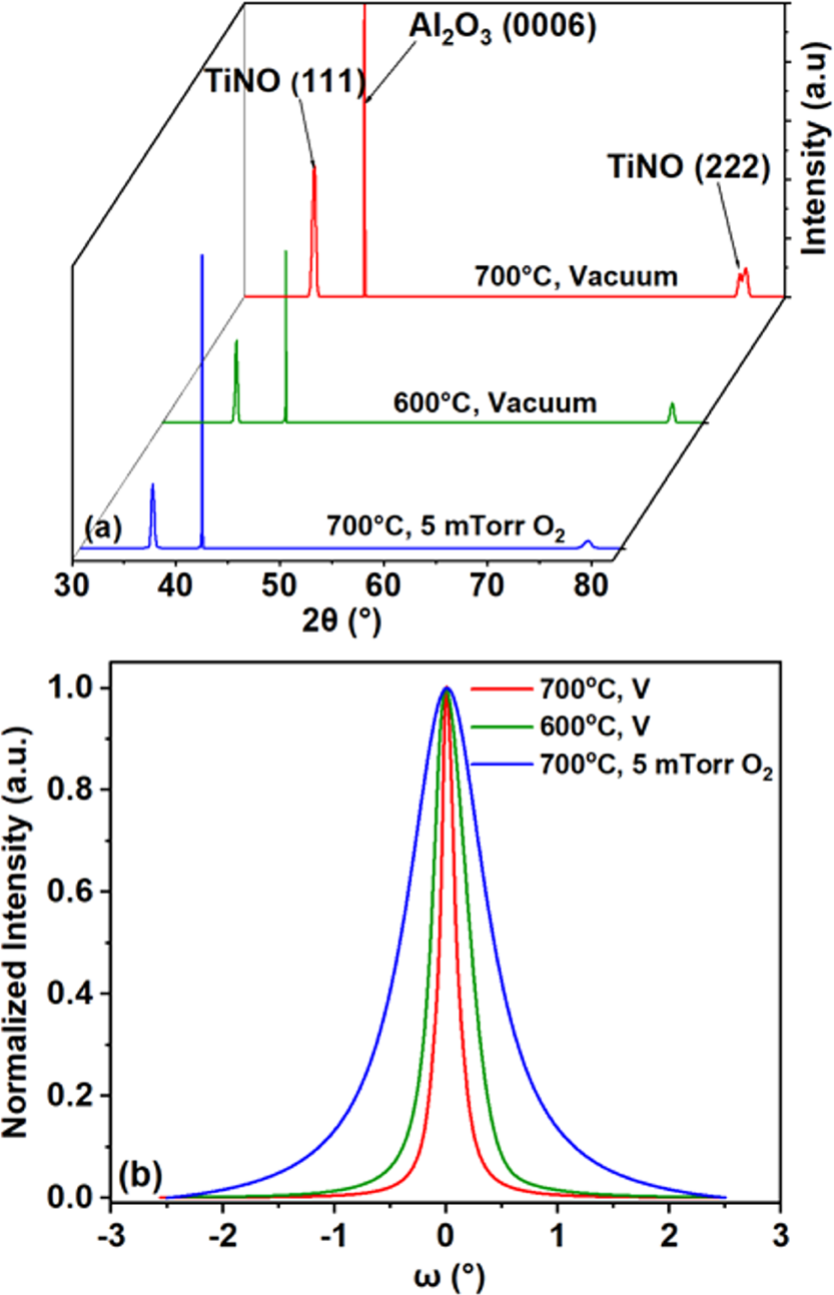
(a) X-ray diffraction patterns of TiNO deposited at different temperatures in vacuum and a 5 mTorr oxygen pressure, and (b) omega curves of the films whose XRD patterns are shown in (a).

The XRD patterns recorded from TiNO thin films grown on *c*-plane single-crystal sapphire substrates in a vacuum of 1.5 × 10^–6^ Torr with no intentional addition of oxygen at 600 and 700 °C and in an oxygen pressure of 5 mTorr at 700 °C are shown in Figure 2a. All the films exhibit characteristic peaks of a rocksalt TiN crystal structure marked by a first harmonic (111) peak at ∼36.73° and a second harmonic (222) peak at ∼77.62°.^51,52,65^ The sharpness of the XRD peaks is close to that of the single crystal substrate peak (41.72°). As seen in Figure 2b, a high degree of crystallinity of these films is reflected from the Omega Rocking Curves (ORCs) recorded for the (111) peaks; the fwhms of the rocking curves of TiN films deposited in vacuum at 700 and 600 °C were found to be 0.154° and 0.364°, respectively, while the fwhm for the TiN thin film deposited in 5 mTorr of O_2_ at 700 °C was considerably larger (0.873°); this larger fwhm value could be due to a larger fraction of TiO_2_, which is mostly amorphous at this growth temperature. As a reference, the rocking curve fwhm of the sapphire (0001) peak was recorded to be 0.030°. While these films are textured and highly crystalline, there is a noticeable shift in the peak positions for all three films with respect to pure bulk TiN material.^66^ The peak position shift is largest for the 600 °C and least for the vacuum, 700 °C sample. The shift in the peak positions is explained by the partial oxidation of TiN to TiNO, which also has the rocksalt crystal structure but with a smaller cell parameter. The lattice constants of these films, calculated from the XRD-measured *d*-values, are 4.235 Å (vacuum, 700 °C), 4.189 Å (vacuum, 600 °C), and 4.206 Å (5 mTorr oxygen, 700 °C). A decrease in the film lattice constant with a reduction in the deposition temperature and an increase in oxygen deposition pressure is thought to be associated with the smaller ionic radius of O^2–^ (1.42 Å) than that of N^3–^ (1.71 Å).^65,66^ The film thickness and density of TiN and TiNO films were estimated by using X-ray reflectometry (XRR) and theoretical fitting of the data. The results obtained are presented in Table S1 and Figure S1. The thickness values obtained using the XRR were used to calculate the resistivity of these films, which are also listed in Table S1. The surface roughness of these films was found to be in the range of 1–3 nm (RMS roughness), which was estimated using atomic force microscopy (AFM) (Figure S2).

**Figure 3:**
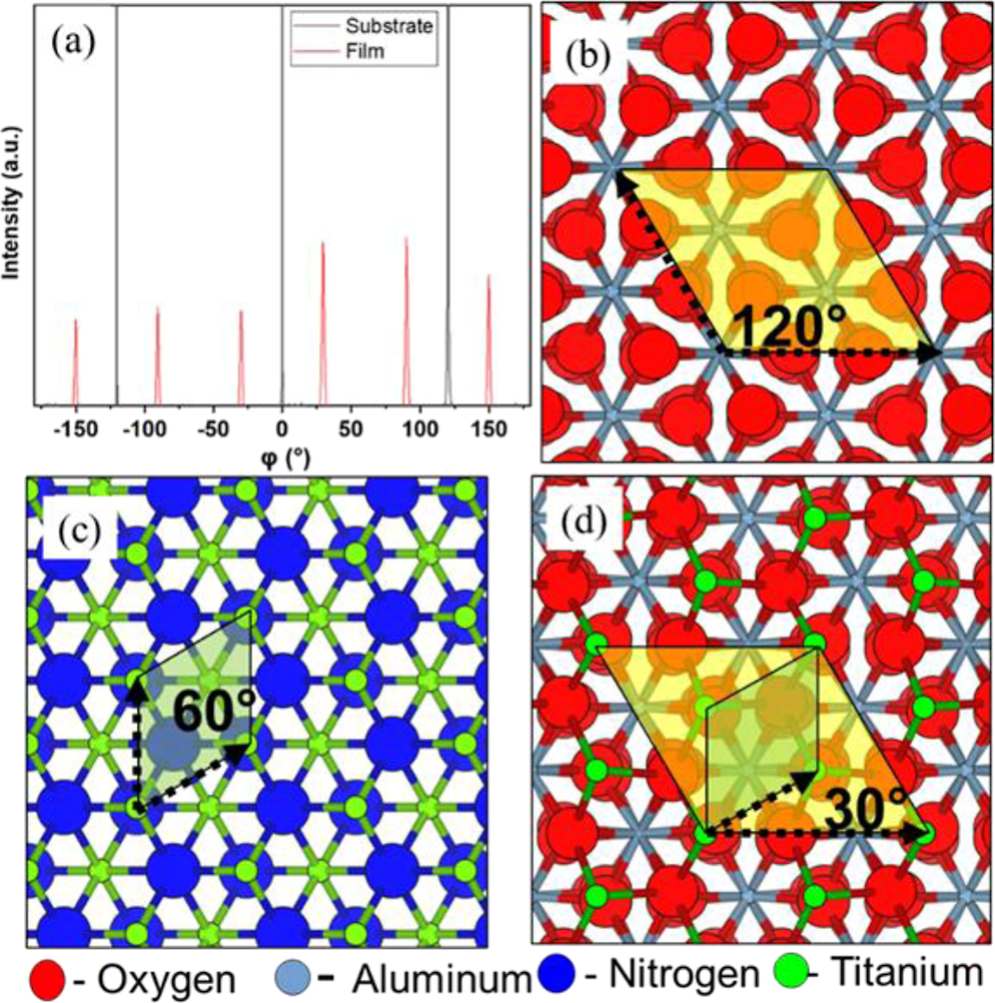
(a) φ-Scan of a vacuum, 600 °C TiNO-film-sapphire structure showing a 30° rotation of the (111) film plane with respect to the (0001) plane of the substrate. (b) 2D primitive unit cell in the (0001) plane of sapphire, (c) 2D primitive unit cell in the (111) plane of TiN, and (d) 30° rotational matching of three 2D unit cells of TiN with one 2D unit cell of the sapphire substrate.

Shown in Figure 3a is the phi-scan recorded to establish the in-plane epitaxial relationship between the film and substrate with the rotation axis perpendicular to the film surface determined as (111)_TiNO_//(0001)_Al_2_O3_ and <110̅>_TiNO_//< 1010̅> _Al_2_O_3__.^29,51,66,67^ The observed reflections are in TiNO(200) and Al_2_O_3_(1012̅) planes.

For reference, projections of the atomic arrangement of the (0001) Al_2_O_3_ and (111) TiN planes are provided in Figure 3b,c, respectively. The Al_2_O_3_ structure coordinate file was obtained from Materials Project mp-1143.^68^ Atomistic renderings were produced using VESTA.^69^ The reflection of TiNO film peaks spaced at 60° in the ϕ-scan is a characteristic of 6-fold symmetry (200 orientation has 3-fold symmetry; the additional peaks arise from the other possible orientations of TiN, which is rotated with minus 30° in Figure 3d), while the reflection of Al_2_O_3_ film peaks spaced at 120° in the ϕ-scan is a characteristic of 3-fold symmetry. The ϕ-scan shows a ±30° rotation around the (111) film, which is parallel to the (0001) plane of the substrate.

The 30° rotation is explained using 2-D oblique primitive unit cell structures of sapphire (Al_2_O_3_) and film (TiN) materials, as illustrated in Figure 3d. For illustration purposes, the model sapphire surface is assumed to be oxygen-terminated. This structure is representative of a Gibbsite-like Al(OH)_3_ surface layer commonly found on sapphire exposed to ambient conditions, which has been dehydrated due to exposure to elevated temperature during processing in vacuum before deposition.^70,71^ It should be noted that in the absence of environmental exposure, sapphire is typically Al-terminated.^71^ The film growth is assumed to begin with a titanium layer with titanium atoms located over hollow sites on the sapphire surface. The exact nature of the sapphire surface and TiN interface will be the subject of future theoretical studies. The 2D lattice parameters of sapphire and TiN are 4.8 and 3.0 Å, respectively. A 30° rotation of the TiN 2D cell with respect to the 2D cell of the sapphire substrate gives a 2D lattice parameter match of ∼8.1%, which is less than the maximum accepted value of lattice mismatch of 10% that still allows epitaxial film growth. In this calculation, three 2D unit cells of TiN are matched with one 2D unit cell of sapphire (Figure 3d) via domain epitaxy.^50,51,67^ This rotation is explained in terms of the TiNO layer formation and straining at the interface with the substrate^29,66,72−74^ due to alignment and rotation of the nitrogen or oxygen atoms of the TiNO film with respect to the oxygen atoms of the Al_2_O_3_ substrate leading to an energetically preferred orientation. Similarly, a minus 30° rotation is also possible for TiN cells due to substrate symmetry.

**Figure 4:**
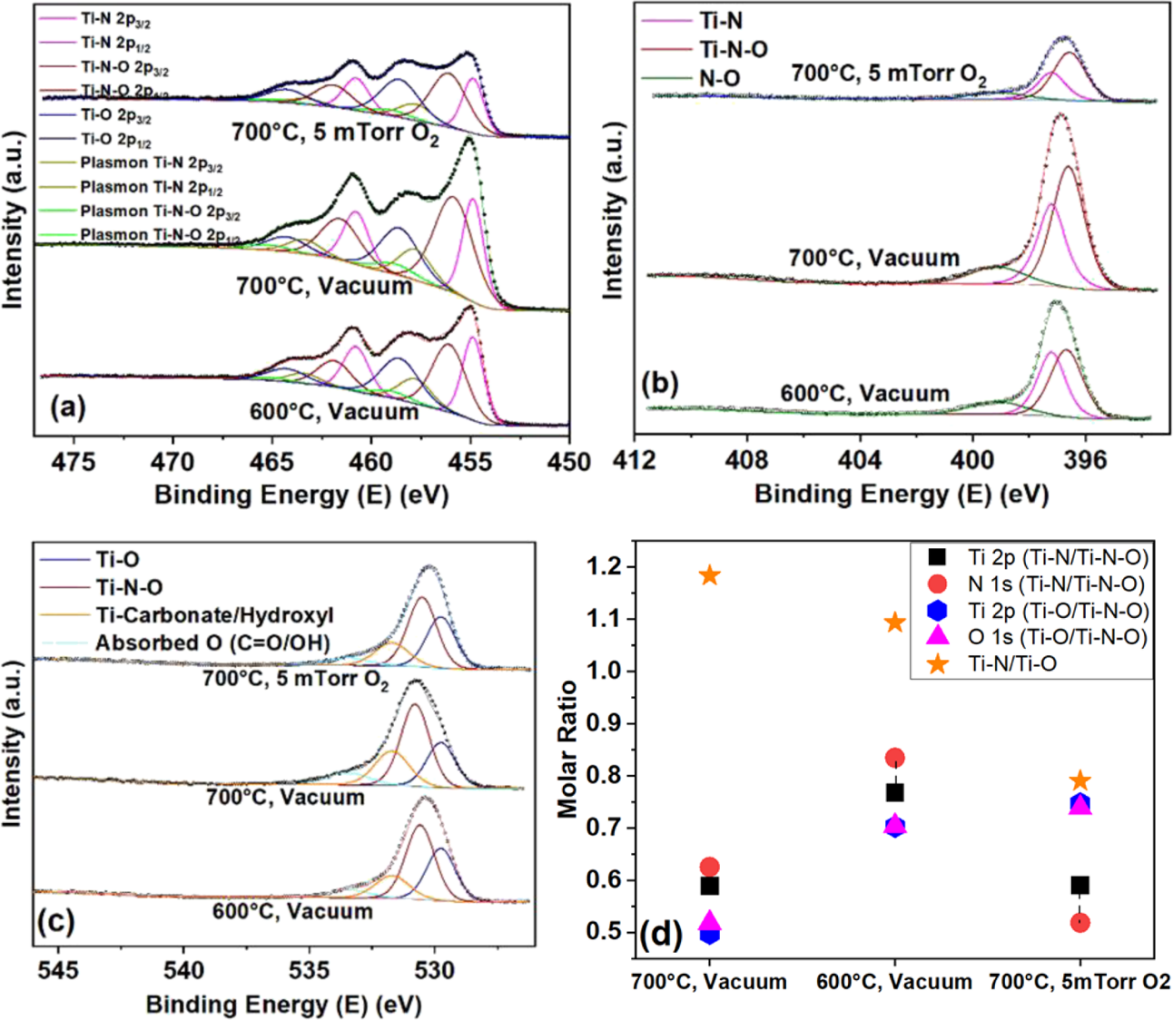
XPS high-resolution spectra after 120s Ar sputtering of (a) the Ti 2p core level, (b) the N 1s core level, and (c) the O 1s core level. (d) Molar ratio comparison across the various spectra (accuracy of fit/error bar).

The Ti 2p core-level XPS spectra are shown in Figure 4a. It is a common practice in the XPS analysis of the Ti 2p spectrum to assign unresolved and overlapping XPS features of differing shapes and intensities as satellite, shoulder, or hump.^75−77^ For the deconvolution of the Ti 2p XPS spectra, a feature between the 456–459 eV is defined as a shakeup satellite.^75^ A shakeup is caused by the transitions in the valence band that occur with a reduction of the kinetic energy from the emitted transitional metal band electron;^76^ others have argued against the in situ formation of titanium dioxide and other oxynitride species.^78,79^ However, these approaches neglect the likeliness of the formation of various oxidation states of titanium that are common among transitional metal XPS spectra.^80,81^ As seen in Figure 4a, a doublet of Ti 2p peaks is noticed in the XPS spectra, which is attributed to spin–orbit coupling between Ti 2p_3/2_ and Ti 2p_1/2_. In our analysis, Ti 2p spectra were deconvoluted to characterize Ti–N, Ti–N–O (as well as their plasmonic features), and Ti–O (TiO_2_) species in all three samples, as shown in Figure 4a. From the XPS spectra measured for the TiNO film deposited under various conditions, the peak position and fwhm of TiN 2p_3/2_ and 2p_1/2_, TiNO 2p_3/2_ and 2p_1/2_, and TiO_2_ 2p_3/2_ and 2p_1/2_ were determined (as seen in Figure S3 and Tables S2 and S3) which match well with those reported in the literature.^35,43,61,75−85^ The variation in the fwhm of TiO_2_ from the values reported in the literature is believed to arise due to the deviation of TiO_2_ stoichiometry (1.04 eV FHWM at a 20 eV pass energy at Ti 2p_3/2_).^81^ The appropriateness of our fitting approach is illustrated by the fact that the interval between the 2p_3/2_ and 2p_1/2_-doublet peaks remained unchanged for TiN (5.9 eV) and TiO_2_ (5.7 eV) for all of the samples (Table S2). In contrast, this interval for TiNO varied from 5.72 to 5.82 as a function of different N to O ratios caused by different film deposition conditions (Table S2).

The effect of the spontaneous surface oxidation when the samples are exposed to ambient conditions is visible in the spectra collected from as-received samples, where the oxygen content was high and almost independent of the deposition conditions. This is why we cleaned the surface with a low-energy Ar ion beam (600 eV) for several tens of seconds to remove the surface contamination layer. Once this surface layer (thickness less than 1–2 nm) was removed, the oxygen and nitrogen concentrations showed a strong dependence on the deposition conditions. The XPS data shown in this article were collected under these conditions. Since the vacuum during XPS data acquisition was of the order of 10^–10^ mbar, the oxidation of the analyzed surface was negligible during the time duration of the data acquisition (several minutes). The distance between the Ti 2p_3/2_ and Ti 2p_1/2_ peaks was fixed for TiN and TiO_2_ compounds and allowed to slowly change for TiON due to a small change in the O and N concentrations. The ratio of the Ti 2p_3/2_ and Ti 2p_1/2_ peaks was also fixed at the reference value of ∼2/1. The O 1s peak has three main components: one for TiO_2_, one for TiON, and one for carbonate/hydroxylate; since the fitting is excellent and consistent for all samples, we think there is no need to add extra peaks for the presence of hypothetical TiO or Ti_2_O_3_ compounds.

In Figure 4b, the core-level N 1s spectra acquired for each sample have been deconvoluted into three peaks corresponding to titanium–nitrogen–oxygen bonding in TiNO, titanium–nitrogen bonding in TiN, and unattached nitrogen–oxygen referred to as chemisorbed/trapped nitrogen, respectively.^35,43,75^ A mixture of surface effects (substitutional and interstitial nitrogen), which represents a variety of species such as N–O, N=O, and other nitrates, accounts for the large fwhm as seen in Table S4 and Figure S4. The molar fractions of TiN and TiNO calculated from the deconvoluted N 1s peak agree with the relative molar fractions of TiN and TiNO calculated from the deconvoluted Ti 2p peak (Figure 4d and Table S4). Similarly, the core-level O 1s spectra have been deconvoluted into three peaks (Figure 4c) corresponding to titanium–oxygen–nitrogen bonding in TiNO, titanium–oxygen bonding in TiO_2_, and (these bonding energies are significantly lower than that for TiO_2_) Ti-carbonate/hydroxyl bonding and adsorbed oxygen which represents a variety of species such as C–OH, C–O, C=O, N=O, and other atmospheric vapor oxides^35,43,75,86−90^ which accounts for the large fwhm as seen in Figure S5 and Table S5. Now, the molar fractions of TiNO and TiO_2_ calculated from the deconvoluted O 1s peak also agree with the molar fractions of TiNO and TiO_2_ calculated from the deconvoluted Ti 2p peak (Figure 4d and Table S3). The XPS results in reference to the elemental composition (Table S6 and Figure S6) were confirmed using non-Rutherford scattering spectrometry (Table S7 and Figures S7 and S8). However, looking at the O 1s peak, there are 3 main components: one for TiO_2_, one for TiNO, and one for carbonate/hydroxylate; the fitting is excellent and consistent for all samples, yet again, there is no need to add extra peaks for TiO or Ti_2_O_3_.

**Figure 5:**
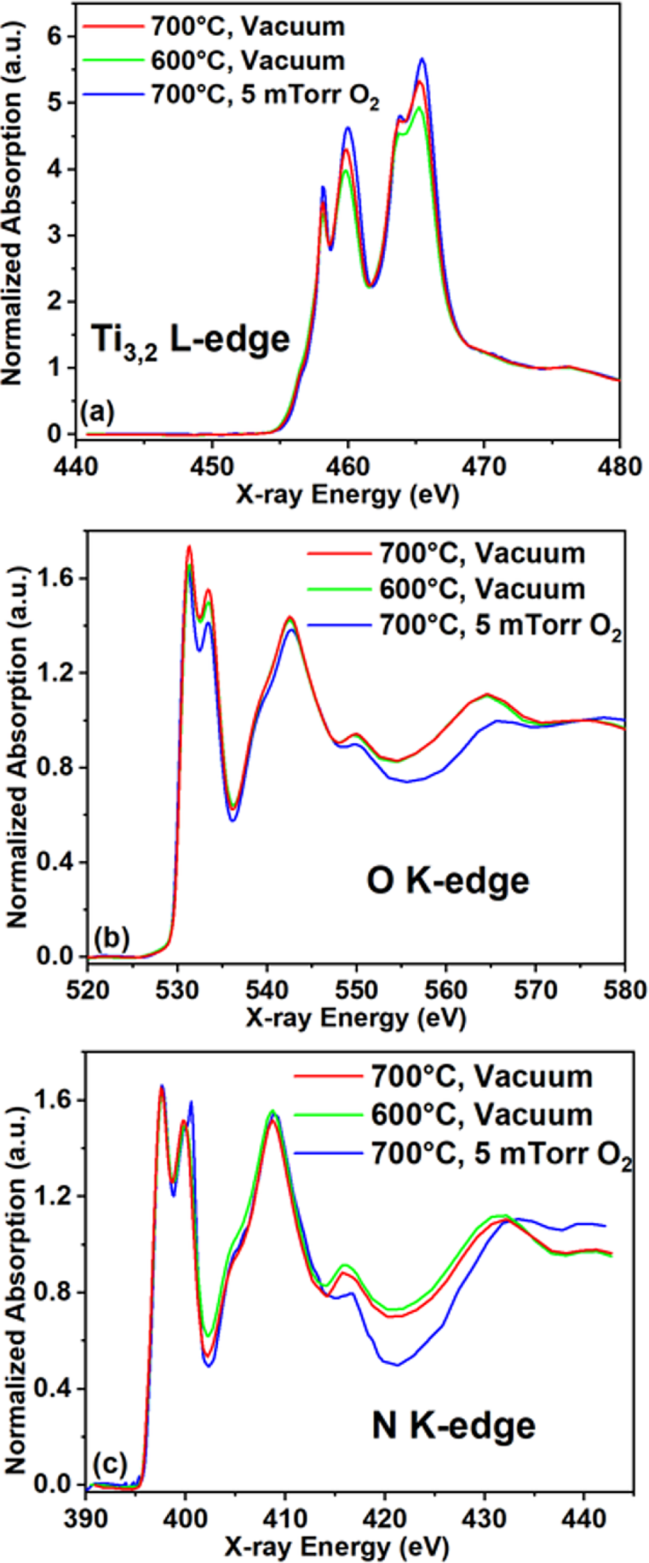
Soft X-ray absorption spectra of the (black) 600 °C-vacuum sample, (red) 700 °C-vacuum sample, and (blue) 700 °C-5 mTorr O_2_ sample at the (a) Ti L_3,2_-, (b) O K-, and (c) N K-edges.

Soft X-ray absorption spectroscopy (XAS) was carried out at the Ti L_3,2_-edge, the O K-edge, and the N K-edge to further understand the chemical structure of the samples. The results obtained are presented in Figure 5. The spectra were acquired in total electron yield (TEY) mode, which is sensitive to the top <10 nm from the surface. The peaks in the Ti L_3,2_-edge spectra (Figure 5a) correspond to the excitation of Ti 2p_3/2_ and 2p_1/2_ electrons to empty Ti 3d states.^91,92^ The spectra shift to slightly higher energies in the order of 600 °C-vacuum → 700 °C-vacuum → 700 °C–5 mTorr O_2_. However, the magnitude of the peaks, normalized to continuum absorption at 480 eV, increases more clearly in this order. This indicates an increase in the average oxidation state of Ti in the surface region of the samples in this order.^93^ This may seem contradictory with the XPS results that showed more oxygen in the 600 °C-vacuum sample than in the 700 °C-vacuum sample. We note that XPS was acquired after Ar^+^ sputtering while XAS was acquired as is in surface-sensitive TEY mode; therefore, the 700 °C sample might be more oxidized on the top surface compared to the 600 °C sample. The O K-edge (Figure 5b) and N K-edge (Figure 5c) XAS shows two peaks at low energy and broader peaks at higher energy levels. We attribute the pair of low-energy peaks to the excitation of O/N 1s electrons to O/N 2p orbitals hybridized with Ti 3d orbitals with t_2g_ and e_g_ symmetry, and the broad high-energy features to O/N 2p hybridized with Ti sp and other higher unoccupied states.^94,95^ The two vacuum samples have similar spectra at the O K-edge and N K-edge, implying a similar average coordination environment around the anions. On the other hand, the 700 °C-5 mTorr O_2_ sample had an altered spectrum, which is consistent with a significantly higher degree of oxidation. The N K-edge of this sample also showed a small peak on top of the e_g_ peak at around 401 eV. This feature had been observed in other studies of oxidized TMNs and assigned to trapped N_2_ molecules generated from the oxidation of TiN.^92,94,96^

### 3.2 Optical Properties

**Figure 6:**
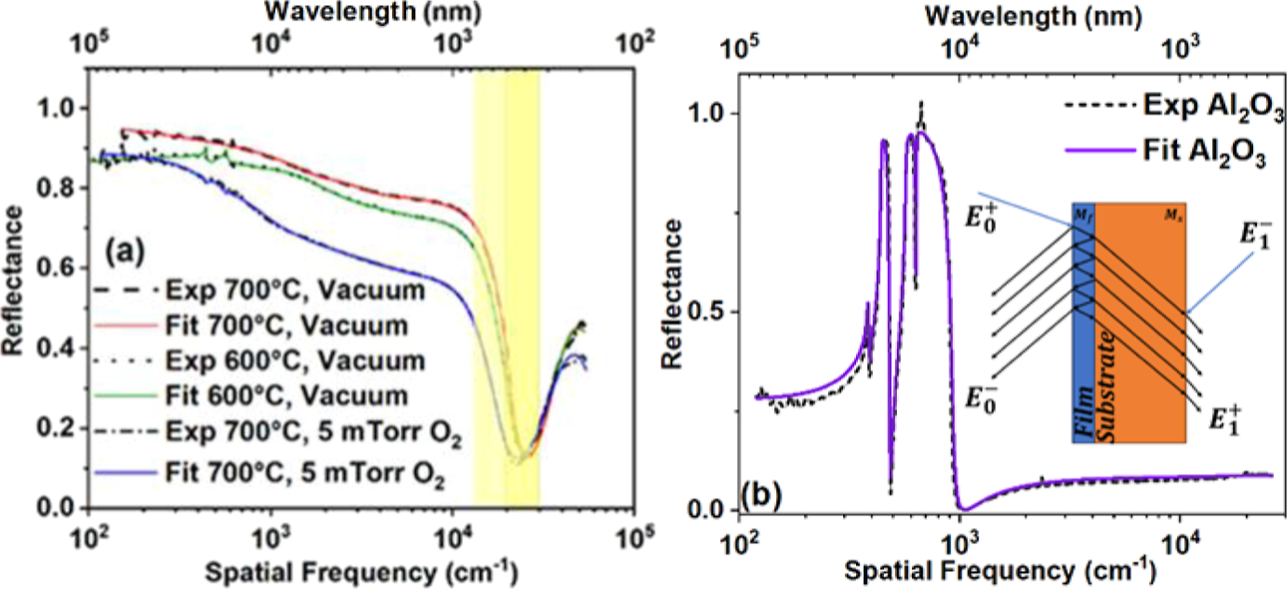
Reflectance spectra and the Lorentz–Drude fit of (a) the various films and (b) sapphire substrate (inset—a model of a thin film (*M*_f_) on an Al_2_O_3_ substrate (*M*_s_), at the air–film, film–substrate, and substrate–air interfaces and four media).

The optical reflectance (*R*) of crystalline TiN films deposited at 600 and 700 °C under high vacuum conditions is shown in Figure 6a as a function of spatial frequency (ν̅ bottom *x*-axis) and wavelength (λ, top *x*-axis). The spatial frequency, also called wavenumber as well as repetency, is a measure of the number of wave cycles per unit distance, i.e., ν̅ = 1/λ. The temporal frequency (ν) with the unit of hertz is the number of wave cycles passing a fixed point in a given time, i.e., ν = *c*/λ = *c*ν̅. The third set of TiN films was deposited in the presence of 5 mTorr of molecular oxygen at 700 °C. The purpose of testing the optical and plasmonic properties of these films was to understand the role of film crystallinity and the role of the oxygen content of TiN films that affects their conductivity. A large reflectance (more than 85%) at a very low excitation energy (<600 cm^–1^ or <75 meV) and well-defined band edge (yellow shaded region) between 22,000 cm^–1^–25,000 cm^–1^, observed for all samples, is consistent with the metallic behavior of these samples. Evidently, the 700 °C vacuum sample has the highest low-frequency reflectance (∼90%), *R*(ω → 0), followed by the 600 °C-vacuum sample (∼85%) and 700 °C–5 mTorr sample (∼82%). The decrease in *R*(ω → 0) values indicates a decrease in the dc-conductivity with oxygen content in the film. Accordingly, the position of the edge, associated with the plasma frequency of the free carriers, shifts slightly to lower energies. The difference in the optical reflectances of these films can be interpreted using the XPS and XRD results. According to the XRD and XPS results, the 700 °C-vacuum TiN sample is less oxidized and more crystalline than the 600 °C-vacuum TiN sample. Less oxygen content in the TiN film and therefore possibly higher carrier concentration may explain the higher energy plasma edge in the 700 °C vacuum film. Higher crystallinity is expected to reduce the electron scattering rate, resulting in higher electrical conductivity and, hence, higher low-frequency reflectance, *R*(ω → 0).^53,97−106^ The intentional addition of oxygen gas during film growth at 700 °C causes the oxidation of highly conducting TiN films to less conducting TiNO films. The XRD results have shown that the 700 °C-5 mTorr sample has the largest fwhm among the three samples. These two effects (poor crystallinity and higher resistivity) are additively manifested in a decrease in the carrier concentration and an increase in the scattering rate.

To clarify and quantify the optical behavior, we performed separate measurements of reflectance on the bare substrate and on the TiN and TiNO film-coated substrates. The analysis involved a model of a thin film of thickness d_f_ on a thick substrate (*d*_s_), therefore including three interfaces (air–film, film–substrate, and substrate–air) and four media, as sketched in the inset of Figure 6b. The first medium and the last medium are semi-infinite with a refractive index of n_0_ = 1. First, measured separately is the reflectance of the substrate using the Lorentz–Drude equation to fit the data and obtain a set of Drude (ω_p_, 1/τ) and Lorentz parameters (ω_j_,*S*_j_,1/τ_j_) for the substrate. The fit was performed using the *dff* routine of the optical data analysis package *datan*, developed at the University of Florida,^107^ and the result is shown in Figure 6b. Using the obtained fitting parameters, we then calculated ε̃_s_, , and further determined the transfer matrix of the substrate *M*_s._ Once the substrate is characterized, we use the *tff* routine for multilayer structures of the same package, seeking to optimize a set of Lorentz–Drude parameters for the film (ε̂_f_) while keeping those of the substrate fixed until the measured reflectance *R*_meas_ (ω) is best reproduced. The results of the multilayer structure fit are shown for each sample in Figure 6a. It can be seen that the fit worked well for all three samples.

**Figure 7:**
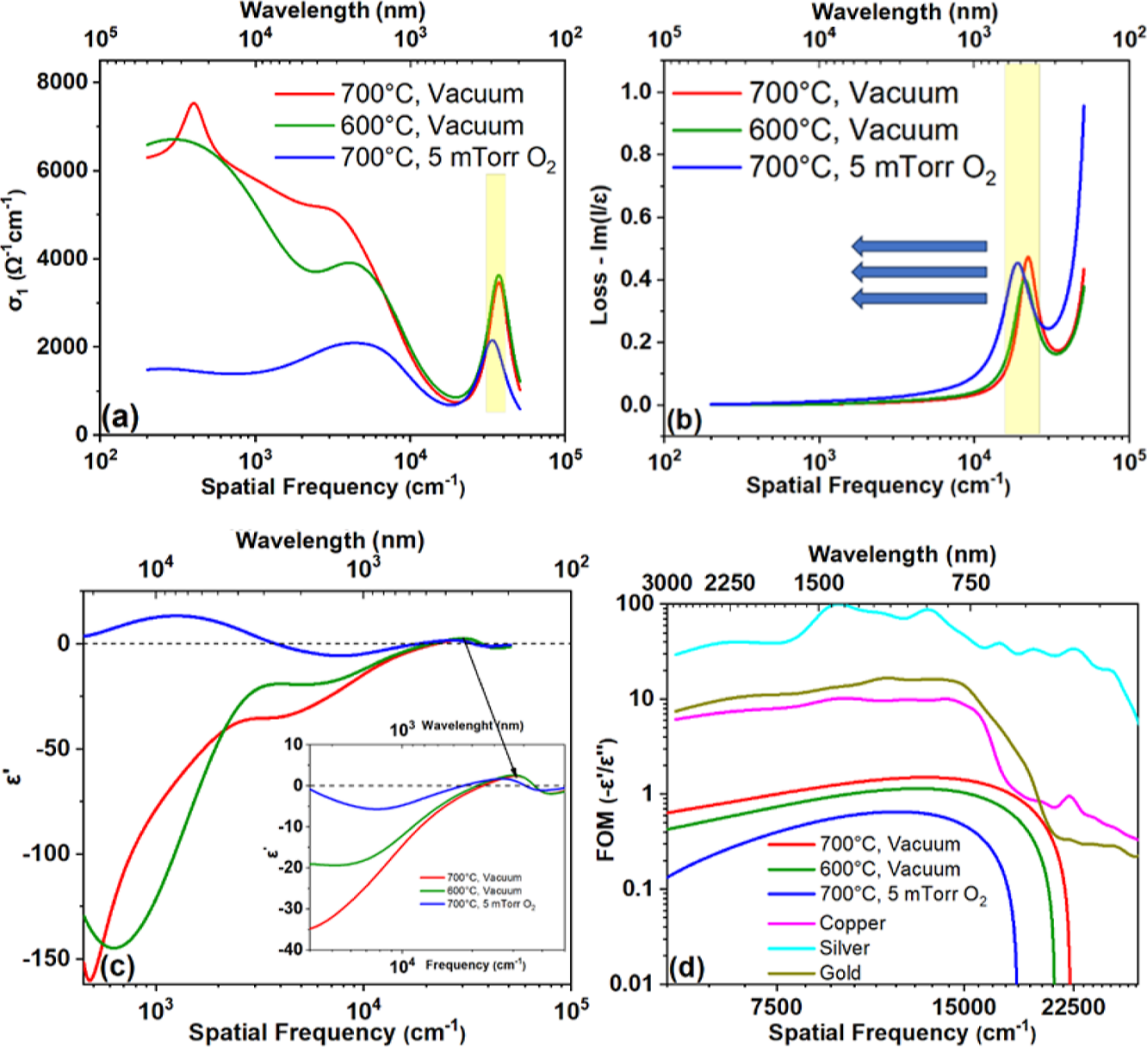
(a) Optical conductivity spectra from the Lorentz–Drude fit of various films; (b) loss function ; (c) real part of the dielectric function ε_1_(ω). (d) FOM – ε′/ε″ of the various films (compared with gold, copper, and silver).^113^

One of the main outcomes of the multilayer approach is that the individual complex dielectric function of film ε̂_*f*_ is extracted from which other optical functions can be calculated (Figure S9). Figure 7a shows the real part of optical conductivity  for all samples. The dc-conductivity σ_DC_ = σ_1_ (0) confirms the qualitative trend with the film oxidation observed from direct measurements of the reflectance. At the same time, it can be noted that the reflectance values are more than double the values obtained if the substrate effect is neglected, which agrees well with the direct DC-transport measurements. The dominant zero-frequency (Drude) peak and the strong absorption at high frequency between 33,000 and 37,000 cm^–1^ (4.09–4.58 eV) are observed; two additional spectral weights below the absorption band of TiN^53^ at the mid-infrared absorption 400 cm^–1^(0.05 eV) and 4000 cm^–1^ (0.5 eV) are also observed. To further confirm the metallic character of the TiNO films, their loss function, , was studied as a function of plasma frequency. A metallic film usually shows a peak at the plasma frequency, the width of which is related to the scattering rate of the free carriers. Figure 7b clearly shows a peak between the 19,000 cm^–1^ and 22,000 cm^–1^ (2.36–2.73 eV), the position of which shifts to low excitation energy (related to an increase in the bandgap) as the TiN films get oxidized as shown by the arrow in the figure. The peak width of each sample also broadens as a function of the plasma frequency of free carriers present in the various samples. As observed from the reflectance band edge, there is an indication of the reduction in concentration and enhanced scattering rate that could be attributed to the decrease in the crystallinity of TiN films. The surface chemistry studied carefully using XPS and XAS points out that as the O/N ratio in TiNO films increases (i.e., as the TiN films get more oxidized), the plasmonic properties of the resulting films, such as reflectivity, the values of negative dielectric constant, and crossover frequency deteriorate. The downgrade in the plasmonic properties with an increase in the O/N ratio is explained on the basis of a decrease in free electron density, which is, in turn, caused by an increase in the bandgap of TiNO with increasing oxygen content.^31,108^ Nevertheless, the TiNO films continue to possess dielectric constants in the film composition range studied in the present research.

Aside from low losses, a large negative dielectric constant ε_1_ is required for plasmonic devices, which has indeed been accomplished, as seen in Figure 7a for all films. The samples obtained in a vacuum have the largest negative values and the largest frequency (energy) of zero-crossing (22247.18 cm^–1^, 21176.53 cm^–1^, and 18652.68 cm^–1^ for 700 °C-vacuum, 600 °C-vacuum, and 700 °C 5 mTorr O_2_, respectively), which is the upper limit for use as plasmonic materials. Interestingly, even the oxidized TiN compounds (TiNO) show feasibility for plasmonic applications, at least in the near to mid-infrared range (>3714 cm^–1^). The figure of merit (FOM), taken as the modulus of real (ε_1_) and imaginary part (ε_2_), for all samples is plotted in Figure 7d as a function of frequency and wavelength. For comparison, we have indicated the peak FOM for Au, Cu, and Ag in the same graph. Au and Ag have nearly an order of magnitude higher FOM with respect to the TiN/TiNO samples. Nevertheless, the tunability of metallicity of TiN/TiNO by means of controlled oxidation and isomorphous phase transformation may offer advantages in terms of swifter conversion of light into heat/electrical energies and may find applications in photothermal and photocatalytic devices.^109−112^ When a visible-light-sensitive semiconducting photocatalyst, such as TiNO, is integrated with TiN plasmonic thin films, a plasmonic Ohmic junction is formed between TiN and the photocatalyst. In this situation, both high-energy (hot) electrons (due to plasmonic transition) and low-energy (cold) electrons (due to interband transition) participate in the charge transfer process, resulting in an enhanced output of photocurrent.^109,112^ In the case of semiconducting photocatalysts integrated with a noble metal plasmonic film, only the hot electrons overcome the Schottky junction barrier height and contribute to the photocurrent.^111^ We plan to carry out this study at a later time.

**Figure 8:**
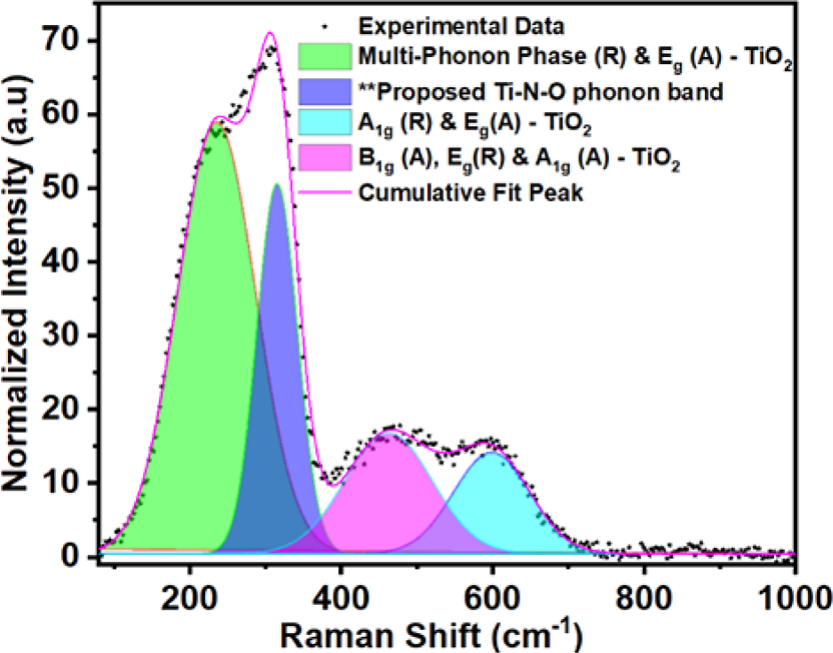
Raman spectra recorded using a 532 nm wavelength laser excitation from the TiNO film deposited at 700 °C–5 mTorr O_2_. Peak fitting was performed for TiNO with respect to the various phonon bands.

As stoichiometric rocksalt TiN is highly symmetrical, its first-order Raman scattering is forbidden, and therefore, no active Raman mode is observed.^114^ However, the substitution of N by O breaks the symmetry, and several Raman peaks are visible, as in Figure S10, which are similar to the bands of TiO_2_ that are marked A and R for anatase and rutile TiO_2_. A broad peak at 316 cm^–1^ (marked by the arrow) is attributed to the Raman scattering in Ti–N–O as there is no literature-reported value of TiO_2_ around this range, as can be seen in Figure 8.^84^

This result compares very well with the XPS Ti 2p deconvolution discussed earlier in this study, where the fractions of TiN and TiNO phases are much higher than the fraction of the TiO_2_ phase. The Ti–N–O bond phonon vibrational modes can either be transversal (T), acoustic (A), longitudinal (L), optical (O),^84,115^ and more specifically, longitudinal acoustic (LA) at ∼320 cm^–1^, transverse acoustic (TA) at ∼235 cm^–1^, and longitudinal optical (TO) at ∼570 cm^–1^.^116−121^ As Raman spectra mirror the density of vibrational states (DVS),^115^ it is observed that the Raman spectra are dominated by an asymmetric band centered at around 320 cm^–1^ and in the lower frequency range by the presence of a phonon band centered at 230 cm^–1^, which signifies the DVS of TiNO films. The higher frequency range of the asymmetrical band is attributed to superposed contributions of the disorder of acoustic phonons and second-order combination of optical and acoustic processes, while the lower frequency range of the asymmetrical band is attributed to the disorder of single phonon and other second-order processes which are not well defined.^122−124^

**Figure 9:**
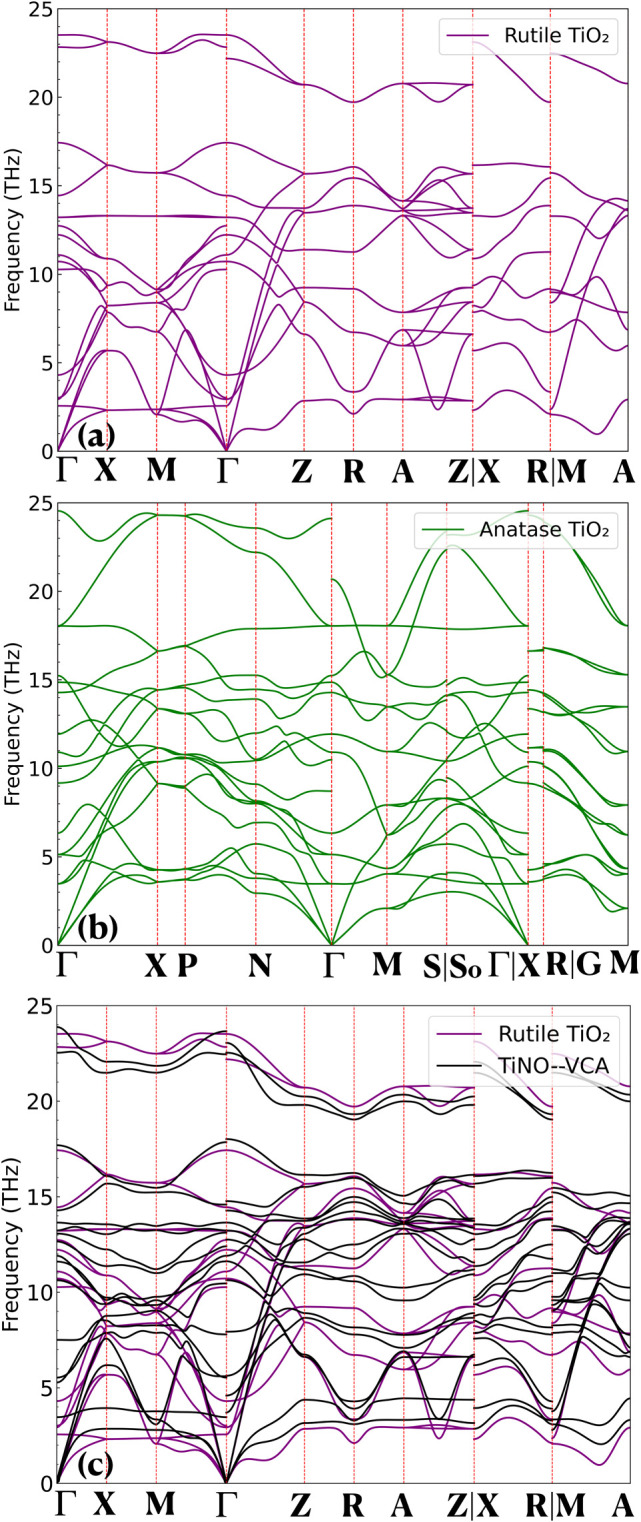
(a) Calculated phonon dispersion of rutile TiO_2_. (b) Calculated phonon dispersions of anatase TiO_2_. (c) Comparison of phonon dispersions between rutile TiO_2_ and virtual crystal TiNO (frequency units in THz).

To corroborate our experimental spectral observations, we calculated the phonon dispersions and Raman active modes for anatase (A) and rutile (R) TiO_2_, as presented in Figure 9a,b. The irreducible representations of rutile (R) TiO_2_ are B_1u_ (85.50 cm^–1^), A_2u_ (100.81 cm^–1^), B_1g_ (143.75 cm^–1^), E_u_ (357.95 cm^–1^), B_1u_ (370.31 cm^–1^), A_2g_ (408.27 cm^–1^), E_g_ (441.35 cm^–1^), E_u_ (482.46 cm^–1^), A_1g_ (582.13 cm^–1^), and B_2g_ (785.37 cm^–1^). For anatase (A) TiO_2_, the irreducible representations are E_g_ (116.03 cm^–1^), E_g_ (171.01 cm^–1^), E_u_ (211.56 cm^–1^), A_2u_ (306.14 cm^–1^), B_1g_ (364.29 cm^–1^), E_u_ (398.50 cm^–1^), B_1g_ (477.95 cm^–1^), A_1g_ (496.26 cm^–1^), B_2u_ (509.05 cm^–1^), and E_g_ (602.68 cm^–1^). Indeed, it is not possible to assign the multiphoton phase-MPP (240 cm^–1^-R) to any of the irreducible representations observed in either anatase (A) or rutile (R) TiO_2_. We thus proceed to calculate the phonon dispersions of TiNO using the virtual crystal approximation. A comparative analysis of the phonon dispersions between rutile TiO_2_ and TiNO is depicted in Figure 9c. Clearly, the incorporation of nitrogen atoms does not significantly alter the phonon dispersion of rutile TiO_2_. However, it results in the emergence of new phonon modes at approximately 7.128 THz (237.65 cm^–1^) at the Gamma point, corresponding to the experimentally observed Multi-Photon Phase-MPP (240 cm^–1^-R).

## 4 Conclusions

The present study reports on the thin film synthesis and precision characterization of titanium nitride and its isostructural oxidative derivative-based negative permittivity, high melting point, and mechanically hard and chemically stable materials beyond commonly employed plasmonic metals (e.g., Au, Ag). The TiNO films deposited at 700 °C in high vacuum conditions have the highest reflectance (*R* = ∼ 95%), largest negative dielectric constant (ε_1_ = −161), and maximal plasmonic figure of merit (FoM = −ε_1_/ε_2_) of 1.2, followed by the 600 °C samples deposited in a vacuum (*R* = ∼85%, ε_1_ = −145, FoM = 0.8) and 700 °C–5 mTorr sample (*R* = ∼ 82%, ε_1_ = −8, FoM = 0.3). An accurate determination of the molar fractions TiN and TiNO in the samples prepared under different oxidation conditions has been reported by observing a near-perfect match in their fractions calculated from the N 1s and Ti 2p peaks in X-ray photoelectron spectroscopy. These measurements are further confirmed by a near-perfect match in the molar fractions of TiNO and TiO_2_ calculated from the XPS O 1s and the Ti 2p peaks. The optical conductivity of these films was analyzed by using a Kramers–Kronig transformation of reflectance and a Lorentz–Drude model; the optical conductivity determined by two different methods agrees very well. The advantages of oxide derivatives of TiN are the continuation of similar free electron density as in TiN and the acquisition of additional features such as oxygen-dependent semiconductivity with tunable bandgap.

## Data Availability

The raw/processed data required to reproduce these findings cannot be shared at this time due to technical or time limitations.

## Supporting Information Available

Experimental methods; XRD and XRR analysis; AFM images; Ti 2p, N 1s, and O 1s XPS fitting parameters; relative molar fraction from the Ti 2p, O 1s and N 1s XPS spectra; XPS elemental and structural composition; Ti 2p, N 1s, and O 1s deconvoluted XPS spectra; XPS depth profile; NRBS data (nitrogen resonance and oxygen resonance; extinction coefficient and refractive index; Raman spectroscopy; and first-principles calculation and lattice dynamics (PDF)
