# Supplementary material for: Optical and Plasmonic Properties of High-Electron-Density Epitaxial and Oxidative Controlled Titanium Nitride Thin Films
Source: J Phys Chem C Nanomater Interfaces. 2025 Feb 3;129(7):3762–74. doi: 10.1021/acs.jpcc.4c06969 (PMC11848926; doi:10.1021/acs.jpcc.4c06969)
Supplement: Supplementary file 1 — jp4c06969_si_001.pdf [file jp4c06969_si_001.pdf]

Supporting Information for Publication

Optical and plasmonic properties of high electron density epitaxial and oxidative controlled titanium nitride thin films

Ikenna Chris-Okoro[1], Sheilah Cherono[1], Wisdom Akande[1], Swapnil Nalawade[2], Mengxin Liu[1], Catalin Martin[3], Valentin Craciun[1,4], R. Soyoung Kim[5], Johannes Mahl[5], Tanja Cuk[6], Junko Yano[5], Ethan Crumlin[5], J. David Schall[1], Shyam Aravamudhan[2], Maria Diana Mihai[7,8], Jiongzhi Zheng[9], Lei Zhang[9], Geoffroy Hautier[9], and Dhananjay Kumar[1]*

[1]Department of Mechanical Engineering, North Carolina A&T State University, Greensboro, NC 27411, USA

[2]Joint School of Nanoscience and Nanoengineering, North Carolina A &T State University, Greensboro, NC, USA 27401

[3]School of Theoretical & Applied Sciences, Ramapo College of New Jersey, Mahwah, NJ 07430, USA

[4]National Institute for Laser, Plasma, and Radiation Physics and Extreme Light Infrastructure for Nuclear Physics, RO 060042, Magurele, Romania

[5]Chemical Sciences Division, Lawrence Berkeley National Laboratory, Berkeley, CA 94720

[6]Department of Chemistry, University of Colorado, Boulders, CO 80309, USA

[7]Horia Hulubei National Institute for Physics and Nuclear Engineering, Măgurele, IF, 077125, Romania

[8]Department of Physics, National University of Science and Technology Politehnica Bucharest, RO, 060042, Romania

[9]Thayer School of Engineering, Dartmouth College, NH, USA 03755

*Email: dkumar@ncat.edu

Supporting Note S1. Experimental

Titanium nitride and titanium oxynitride thin films were deposited on 10 mm×10 mm×0.5 mm c-plane sapphire (0001) substrates using a pulsed laser deposition (PLD) technique. A high-purity (99.99%) TiN target (one-inch diameter, quarter-inch thickness) was used to deposit the TiN/TiNO films by controlling the substrate temperature, oxygen pressure, laser energy density, and laser pulse repetition rate. A KrF laser (Lambda Physik, wavelength 248 nm, pulse duration 30 ns) was used. The PLD chamber was pumped down to a base pressure of <3 × 10^{-6} Torr, and subsequently, the substrates were heated to the desired temperatures. A fixed number of laser pulses of 20,000 was used at a laser frequency of 10 Hz. The thin film deposition experiments were carried out in a 5 mTorr O_2 pressure and under a vacuum of 1.5×10^{-6} Torr; the deposition under high vacuum conditions is referred to as deposition in residual oxygen ambient, i.e., no oxygen added intentionally. The substrate temperatures of 600°C to 700°C were used, keeping all other deposition parameters the same in all experiments.

High-resolution x-ray photoelectron spectroscopy (XPS) was recorded for Ti 2p, N 1s, and O 1s core levels to accurately quantify the oxidized, partially oxidized, and unoxidized phases of TiN, avoiding the common errors frequently encountered right from the data collection to subsequent analysis. A precise quantification of these phases is important in understanding the resulting properties of TiNO compounds formed at higher deposition temperatures and in the presence of oxygen ambient. In this respect, all XPS spectra were recorded under low noise with a reasonable peak-to-background ratio. The caution exercised in the subsequent data analysis involved selecting proper model functions, reducing the number of free parameters by using existing information on well-known doublet splitting and intensity ratios, running the fit procedure with Shirley background subtraction with 50 eV and 30 eV pass energy for survey and high-resolution scans, respectively, to obtain a good signal-noise ratio, and taking only < 7 units residual standard deviation fittings. Also, in XPS analysis and fitting, binding energy, FWHM, and spectra shape are kept identical for the same chemistry. For example, the binding energies, FWHM, and spectra shape obtained

using a set of fitting parameters should be independent of sample preparation conditions for one kind of chemistry, namely, in the present study, TiN and TiO_2 chemistry. However, a variation in these parameters (binding energies, FWHM, and spectra shape) for TiNO using the same fitting precautions used for TiN and TiO_2 with well-established chemistries should be taken to confirm changing chemistry and changing O/N ratio in the various films. The C 1s was used as a reference for binding energies; once the C1s was removed from the samples' surface by sputtering, the BEs of Ti bonded to N in TiN and Ti bonding to O in TiO2 were fixed at the values determined when C1s peak was visible. It should be mentioned that this assignment of the BE was self-consistent in the sense that once the BE of pure TiN was fixed at the reference value, also the BE of TiO2 was found at the reference value. Another important finding is that the total amount of Ti, O, and N measured by XPS was consistent with the values measured by NRBS. The fractions of Ti bonded in TiN, TiON, and TiO2 according to the deconvolution process for the Ti 2p peak were also consistent with the N 1s and O 1s deconvolution process. XAS measurements were carried out at Beamline 7.3.1 at the Advanced Light Source (ALS), Lawrence Berkeley National Laboratory.

The elemental composition of the TiN and TiNO films was also determined using non-Rutherford backscattering spectrometry (NRBS) with $^{4}He^{++}$ ions at 3.043 MeV and 3.7 MeV. Light elements like oxygen (O) and nitrogen (N) exhibit a higher cross-section at these specific energies, enabling their differentiation from the substrate signal. The NRBS measurements were performed under high vacuum (10^{-6} mbar), using a collimated $^{4}He^{++}$ beam extracted from the duoplasmatron ion source of the 3 MV Tandetron accelerator of Horia Hulubei National Institute for R&D in Physics and Nuclear Engineering (IFIN-HH). The alpha particles were detected with a passivated, ion-implanted silicon detector positioned at an angle of 165° relative to the incident beam direction. With a detector diameter of 8 mm and a sample-to-detector distance of 175 mm, the configuration yielded a solid angle of 1.641 msr. The measured energy resolution of the detector was 17 keV. During NRBS, the samples were tilted at 7° relative to the beam direction. The total ion dose was 20 μC per spectrum.

Table S1. Film thickness and density of TiN/TiNO thin film samples deposited at 700°C- vacuum, 600°C-vacuum, and 700°C- 5 mTorr O_2, measured using x-ray reflectometry. The electrical resistivity is also tabulated.

Samples	XRR Thickness (nm) ±.05	Density (gcm^{-3}) ±.04	Electrical Resistivity (μΩcm) ±.05
700°C- Vacuum	300	5.4	190
600°C- Vacuum	312	5.3	407
700°C- 5 mTorr O_2	302	5.0	365

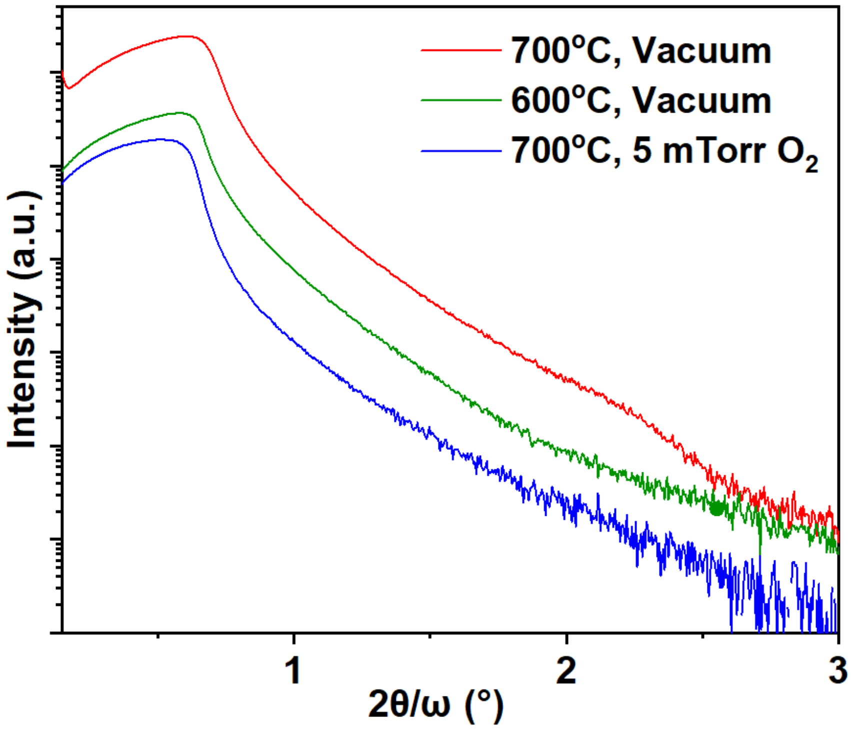

Figure S1. X-ray reflectometry (XRR) plots of TiN/TiNO samples deposited under different condtions; the plots are displaced vertically for better viewing.

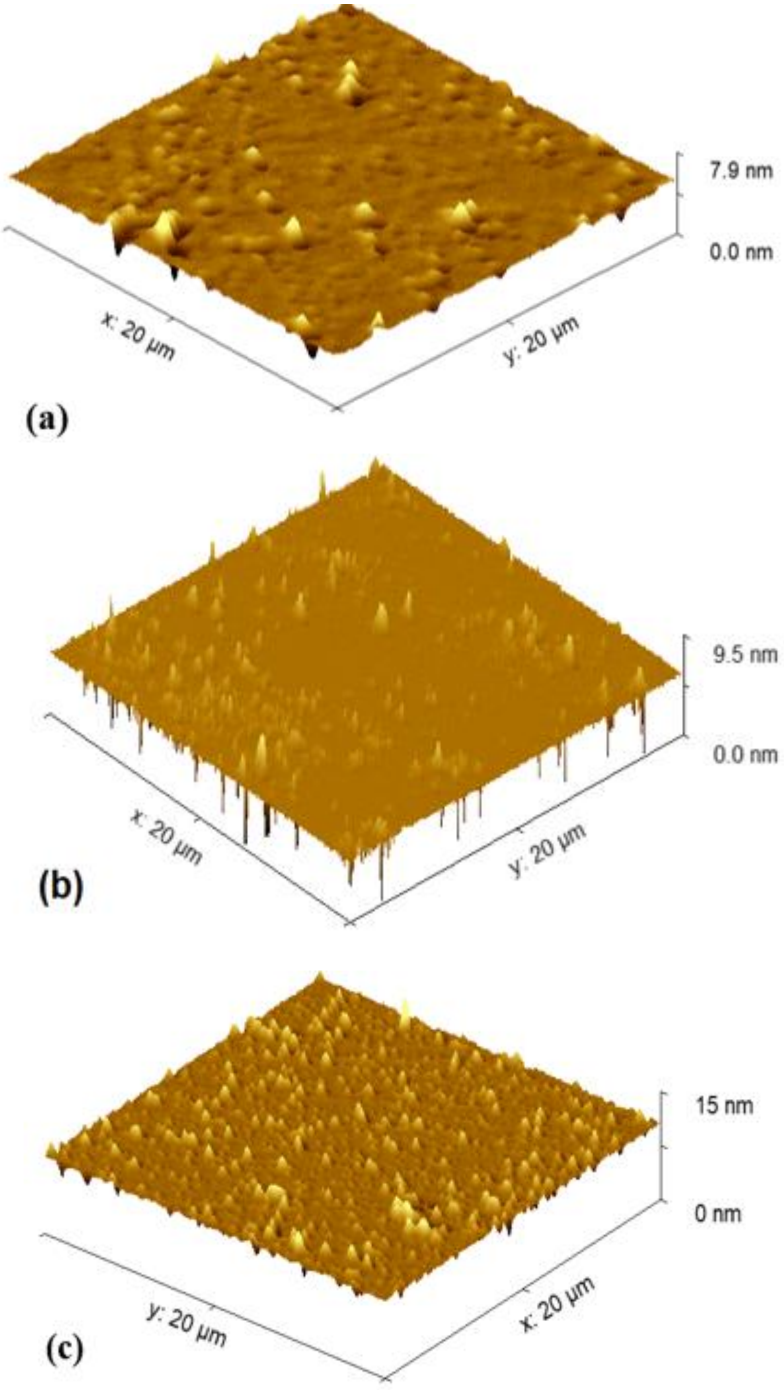

Figure S2. AFM images of (a) 700°C, vacuum (b) 600°C, vacuum (c) 700°C, 5 mTorr O_2.TiN and TiNO films with the root-means roughness square (RMS) roughness of 1.22 nm, 2.54 nm and 3.12 nm, respectively.

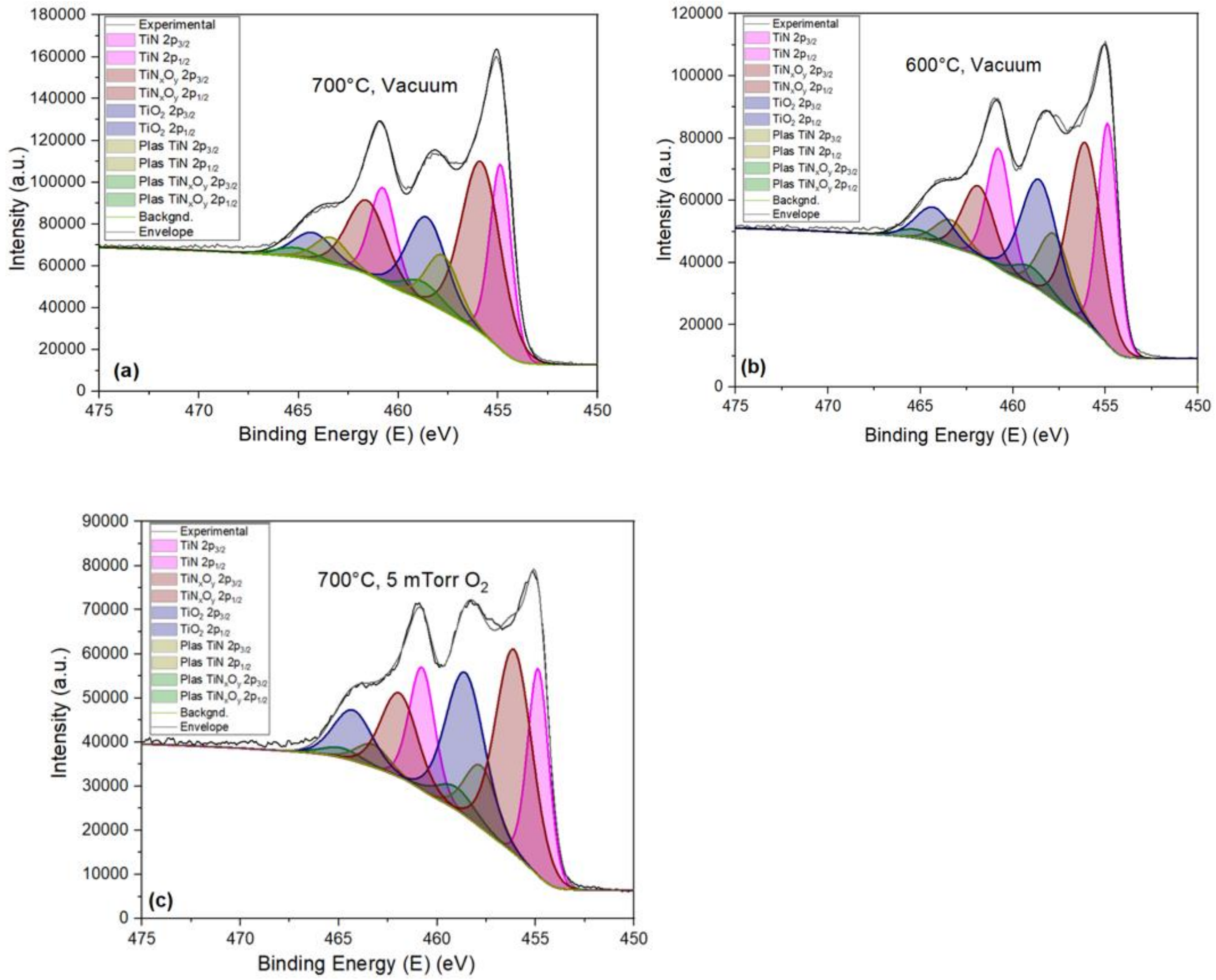

Figure S3. Ti 2p deconvoluted XPS Spectra (a) 700°C, Vacuum (b) 600°C, Vacuum (c) 700°C, 5 mTorr O_2. The Ti $2p_{3/2}$ peak area is reported to be nearly twice the relative area of the Ti $2p_{1/2}$; a deviation in this trend is attributed to a Coster-Kronig effect [1-4].

Table S2. Binding Energies and FWHM of the various species obtained from the fitting of the decovoluted Ti 2p spectra recorded from TiN/TiNO thin film samples deposited at 700°C- Vacuum, 600°C- Vacuum, and 700°C- 5 mTorr O_2. The binding energy difference between Ti $2p_{3/2}$ and Ti $2p_{1/2}$ for TiN, TiNO, and TiO_2 are also listed.

Spectral Species		Binding Energies, eV			FWHM, eV		
		700°C, Vacuum	600°C, Vacuum	700°C, 5 mTorr	700°C, Vacuum	600°C, Vacuum	700°C, 5 mTorr
TiN	$2p_{3/2}$	454.87	454.87	454.87	1.31	1.31	1.31
	$2p_{1/2}$	460.77	460.77	460.77	1.57	1.57	1.57
TiNO	$2p_{3/2}$	455.85	456.09	455.85	2.40	2.09	2.40
	$2p_{1/2}$	461.57	461.87	461.57	2.40	2.09	2.40
TiO_2	$2p_{3/2}$	458.58	458.58	458.58	2.40	2.40	2.40
	$2p_{1/2}$	464.28	464.28	464.28	2.40	2.40	2.40
Plas TiN	$2p_{3/2}$	457.78	457.78	457.78	2.00	2.00	2.00
	$2p_{1/2}$	463.38	463.38	463.38	2.00	2.00	2.00
Plas TiNO	$2p_{3/2}$	458.80	459.04	458.80	2.40	2.40	2.40
	$2p_{1/2}$	465.10	465.24	465.10	2.40	2.40	2.40
TiN Difference		5.90	5.90	5.90			
TiNO Difference		5.72	5.78	5.82			
TiO_2 Difference		5.70	5.70	5.70			

Table S3. Relative molar fraction of various species present in the film deconvoluted from the Ti 2p, O 1s, and N 1s XPS Spectra.

Spectral Species	% Relative Molar Fraction		
	700°C-Vacuum	600°C-Vacuum	700°C- 5 mTorr
TiN (Ti 2p)	28.22	31.08	25.25
TiNO (Ti 2p)	47.94	40.49	42.79
TiO_2 (Ti 2p)	23.84	28.43	31.96
Ti-N (N 1s)	33.67	38.77	29.63
Ti-N-O (N 1s)	53.85	46.46	57.16
N-O (N 1s)	12.48	14.77	13.21
Ti-O (O 1s)	23.35	31.73	32.52
Ti-N-O (O 1s)	45.08	45.1	44.03
Ti-Carbonate/ Hydroxyl (O1s)	22.14	16.19	17.03
Absorbed O (OH-C=O) (O 1s)	9.48	6.98	6.42

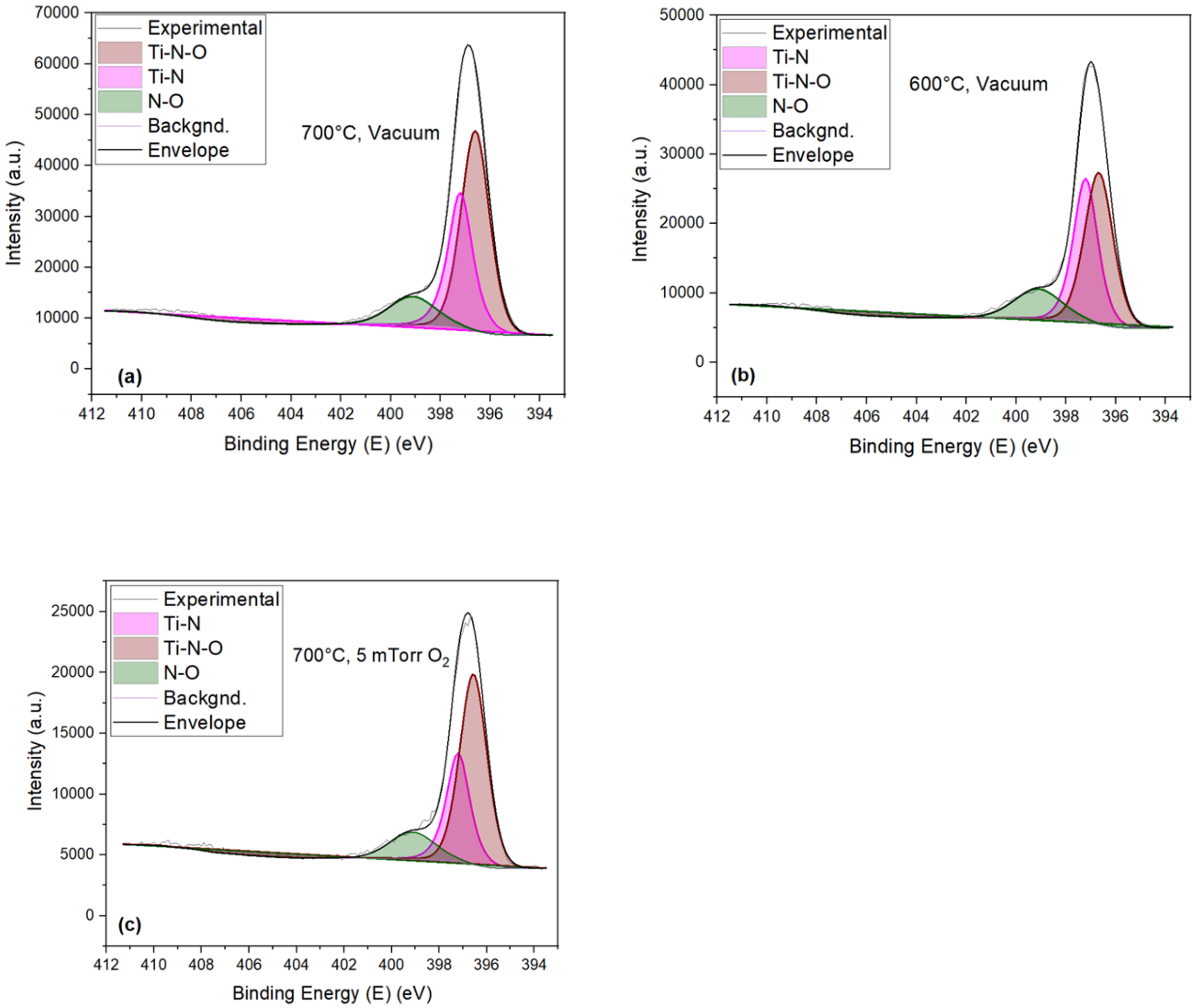

Figure S4. N 1s deconvoluted XPS Spectra for (a) 700°C, Vacuum (b) 600°C, Vacuum (c) 700°C, 5 mTorr O_2

Table S4. Binding Energies and FWHM of the various species obtained from the N 1s fitting of the decovoluted N 1s spectra recorded from TiN/TiNO thin film samples deposited at 700°C- Vacuum, 600°C-Vacuum, and 700°C- 5 mTorr O_2.

Spectral Species	Binding Energies, eV			FWHM, eV		
	700°C-Vacuum	600°C-Vacuum	700°C, 5 mTorr	700°C-Vacuum	600°C-Vacuum	700°C-5 mTorr
Ti-N	397.20	397.20	397.20	1.20	1.20	1.20
Ti-N-O	396.60	396.66	396.68	1.40	1.40	1.40
N-O	399.13	399.13	399.13	2.30	2.30	2.30

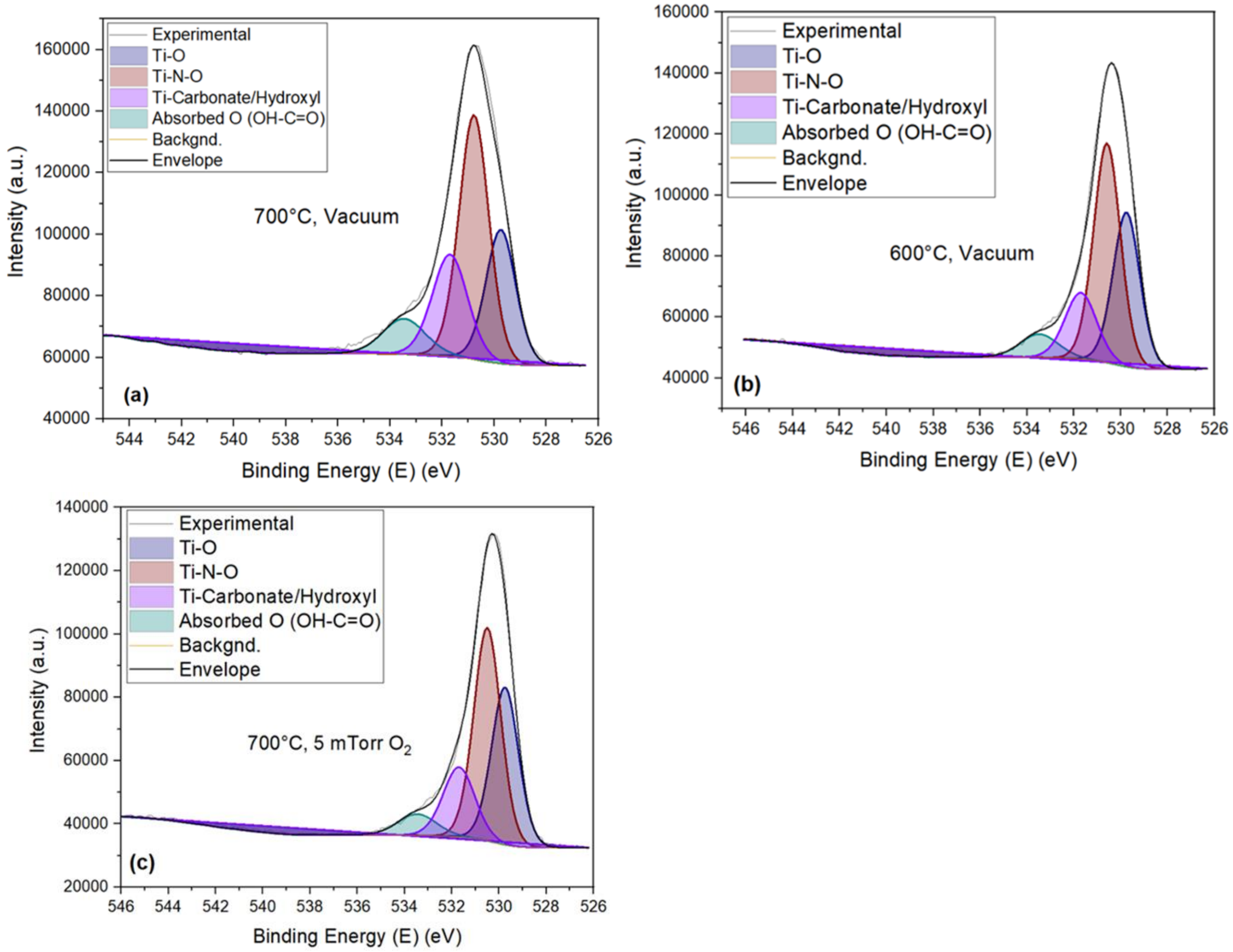

Figure S5. O 1s deconvoluted XPS Spectra for (a) 700°C- Vacuum (b) 600°C- Vacuum (c) 700°C- 5 mTorr O_2 TiN/TiNO films.

Table S5. Binding Energies and FWHM of the various species extracted from O 1s fitting from the decovoluted O 1s spectra recorded from TiN/TiNO thin film samples deposited at 700°C- Vacuum, 600°C-Vacuum, and 700°C- 5 mTorr O_2.

Spectral Species	Binding Energies, eV			FWHM, eV		
	700°C-Vacuum	600°C-Vacuum	700°C-5 mTorr	700°C-Vacuum	600°C-Vacuum	700°C-5 mTorr
Ti-O	529.75	529.75	529.75	1.40	1.40	1.40
Ti-N-O	530.78	530.58	530.50	1.40	1.40	1.40
Ti- Carbonate/ Hydroxyl	531.70	531.70	531.70	1.65	1.65	1.65
Absorbed O (OH-C=O)	533.47	533.47	533.47	2.00	2.00	2.00

Table S6. XPS Elemental compositions.

Samples	Elemental Percent			Chemical Formula
	Ti	N	O	
700ºC, Vacuum	39.42	22.66	37.92	$TiN_{0.57}O_{0.96}$
600ºC, Vacuum	39.10	20.13	40.77	$TiN_{0.51}O_{1.04}$
700ºC, 5 mTorr O_2	37.77	14.03	48.20	$TiN_{0.37}O_{1.28}$

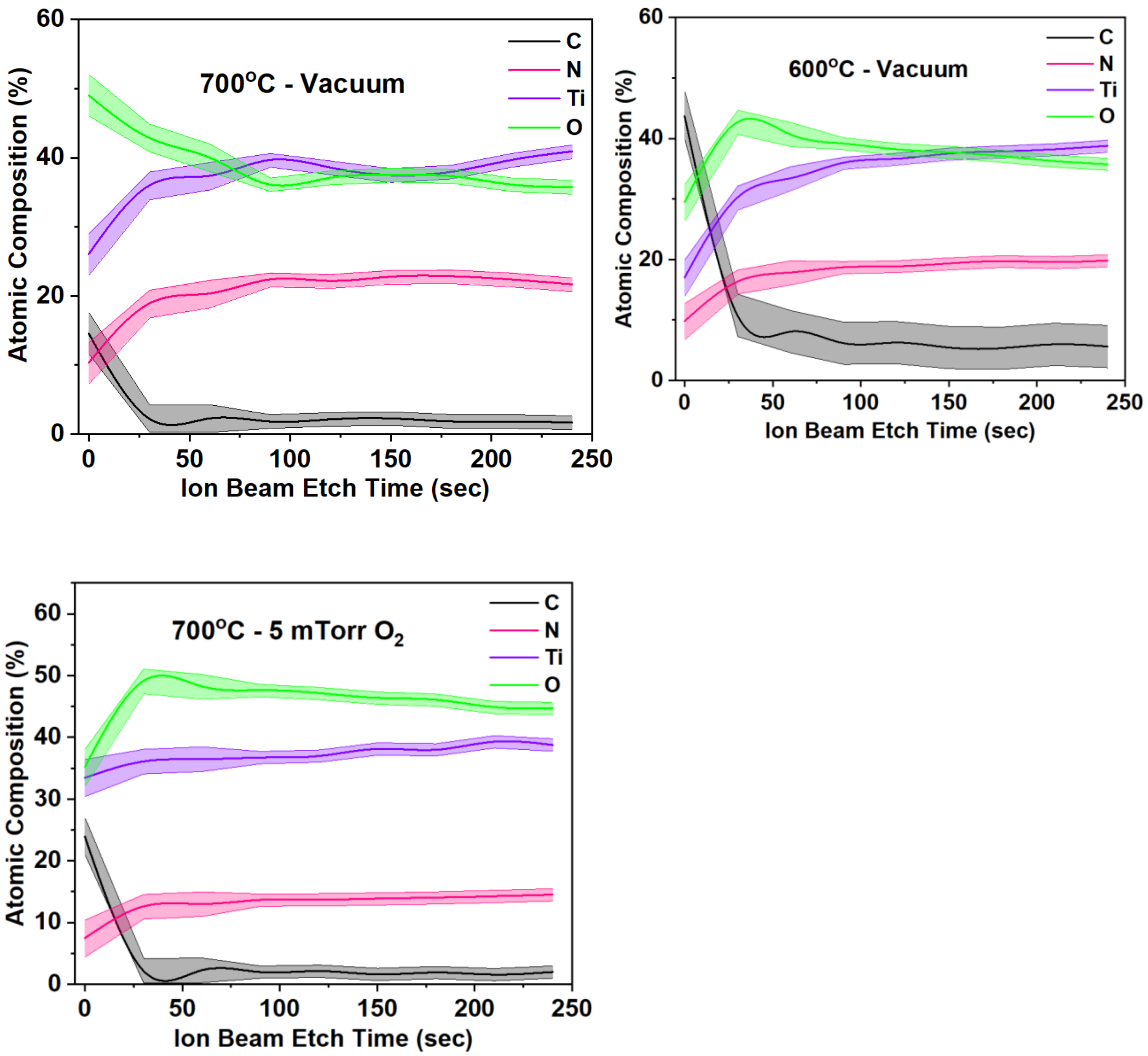

Figure S6. Elemental compositional XPS depth profile recorded at an interval of 30 s showing the constancy of C, N, Ti, O composition for the 700°C- vacuum, 600°C- vacuum, and 700°C- 5 mTorr O_2, samples.

Table S7. Film thickness and stoichiometry of the resulting thin film compounds, estimated from the non-RBS fitting using the SIMNRA software analysis. While the XPS provides the compositional information of the surface, the non-RBS analysis serves as a depth profile analysis for heavier elements in a film lattice containing lighter ones. The error margin of measurement is within a ± 2% range. The decreasing trend ratio of N/O is also seen in the XPS data from the 700°C- vacuum to 700°C- 5 mTorr O_2. The thickness measurements compare well with the XRR analysis. The film composition is also displayed. These non-RBS results compare well with XPS results (Table S6 and Fig. S4) at the surface, confirming the presence of various chemistries across the thickness of the film from a more oxygenated surface to a more nitrogenated bulk.

Sample name	Characterization				
	Layers	Thickness (nm)	Composition		
Nitrogen Resonance 700°C- Vacuum	1	300 nm $TiN_{0.66}O_{0.34}$	N 0.33	Ti 0.50	O 0.17
	2*	500000	Al 0.40	O 0.60	
Nitrogen Resonance 600°C- Vacuum	1	314 nm $TiN_{0.63}O_{0.69}$	N 0.27	Ti 0.43	O 0.30
	2*	500000	Al 0.40	O 0.60	
Nitrogen Resonance 700°C- 5 mTorr O_2	1	302 nm $TiN_{0.46}O_{0.99}$	N 0.19	Ti 0.41	O 0.40
	2*	500000	O 0.60	Al 0.40	

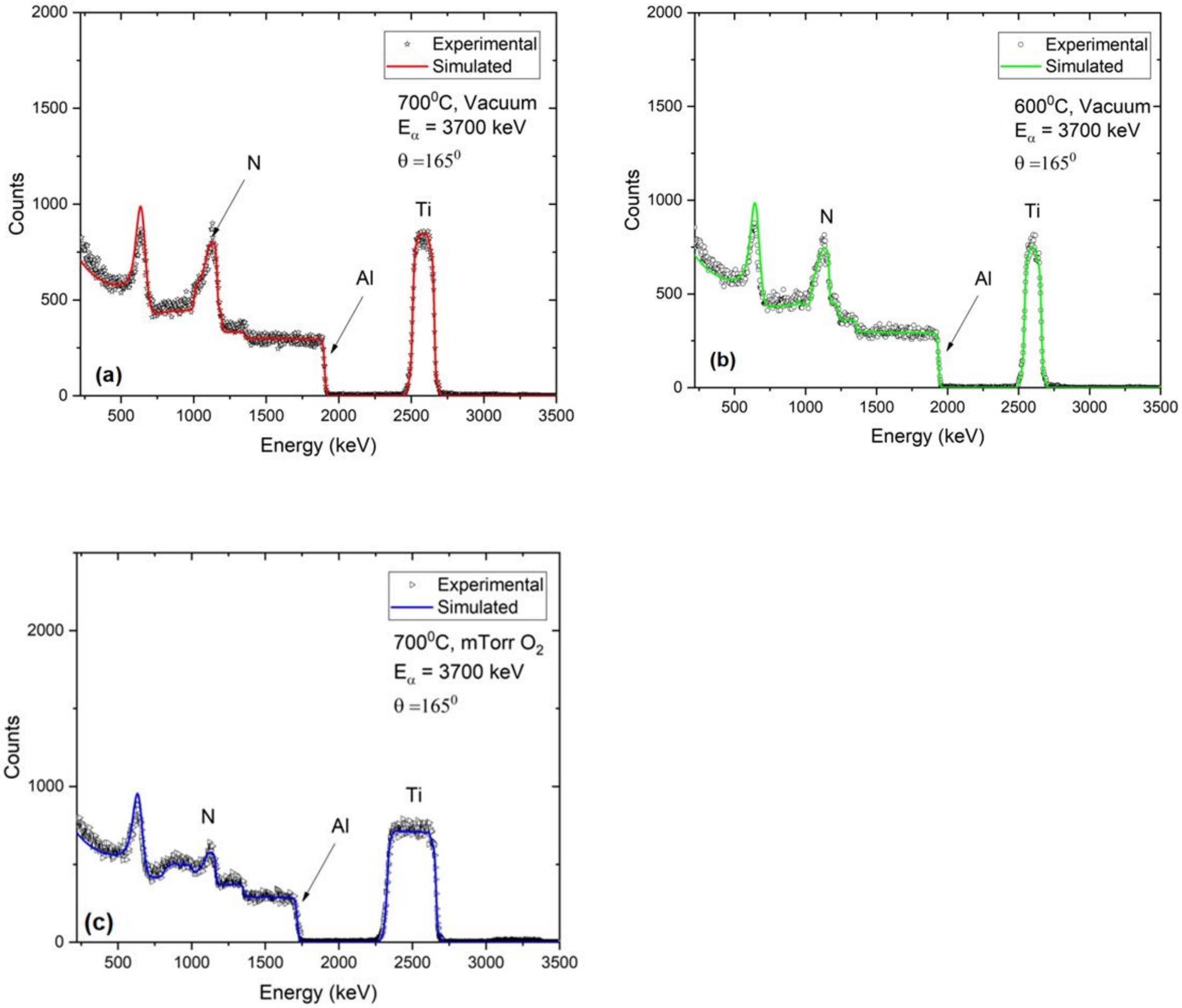

Figure S7. Non-Rutherford Backscattering Spectrometry and fitting for nitrogen resonance for TiN/TiNO films deposited at (a) 700°C- vacuum, (b) 600°C- vacuum, and (c) 700°C- 5 mTorr O_2,

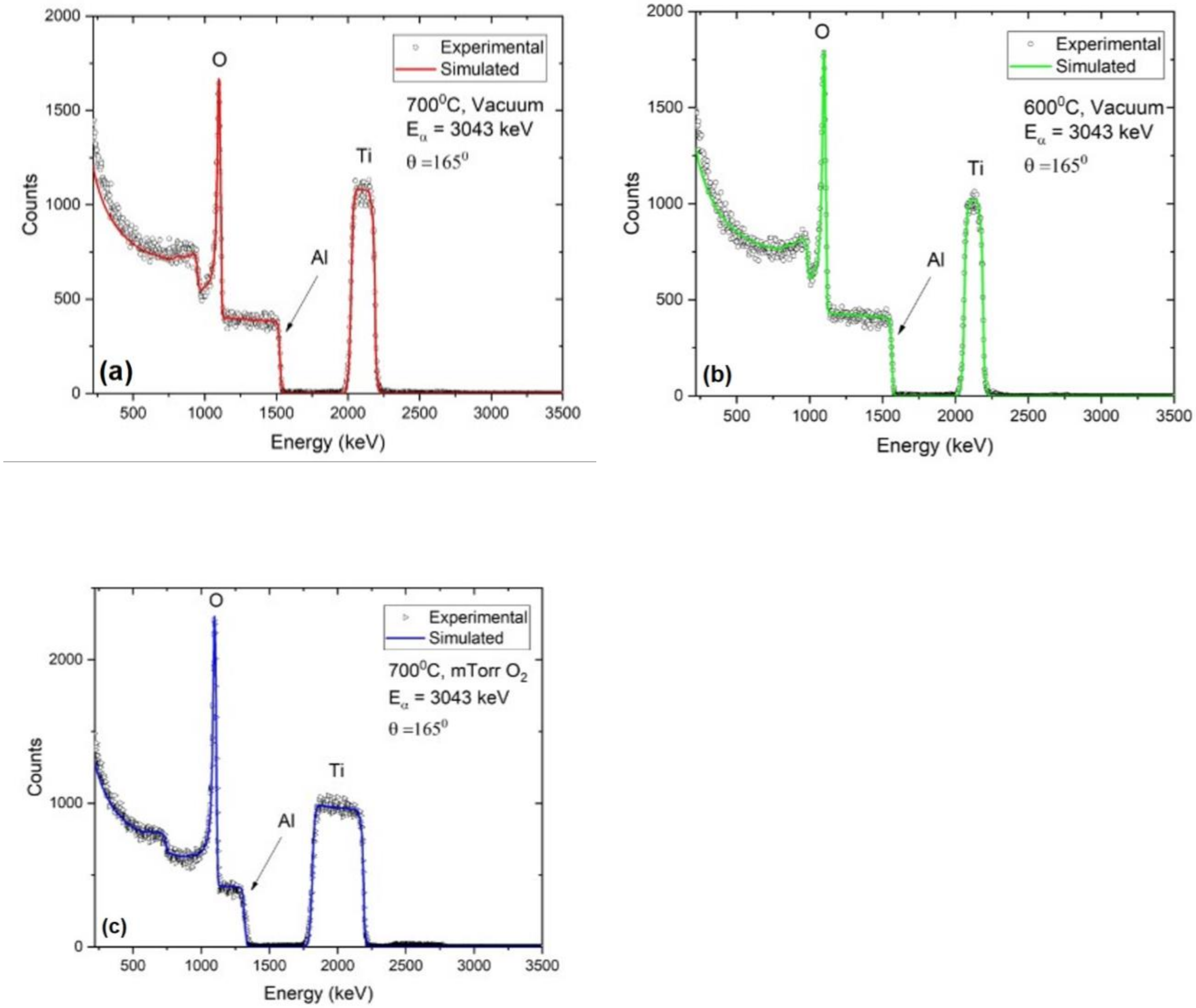

Figure S8. Non-Rutherford Backscaterring Spectrometry and fitting for oxygen resonance for TiN/TiNO films (a) 700°C- vacuum, (b) 600°C- vacuum, and (c) 700°C- 5 mTorr O_2.

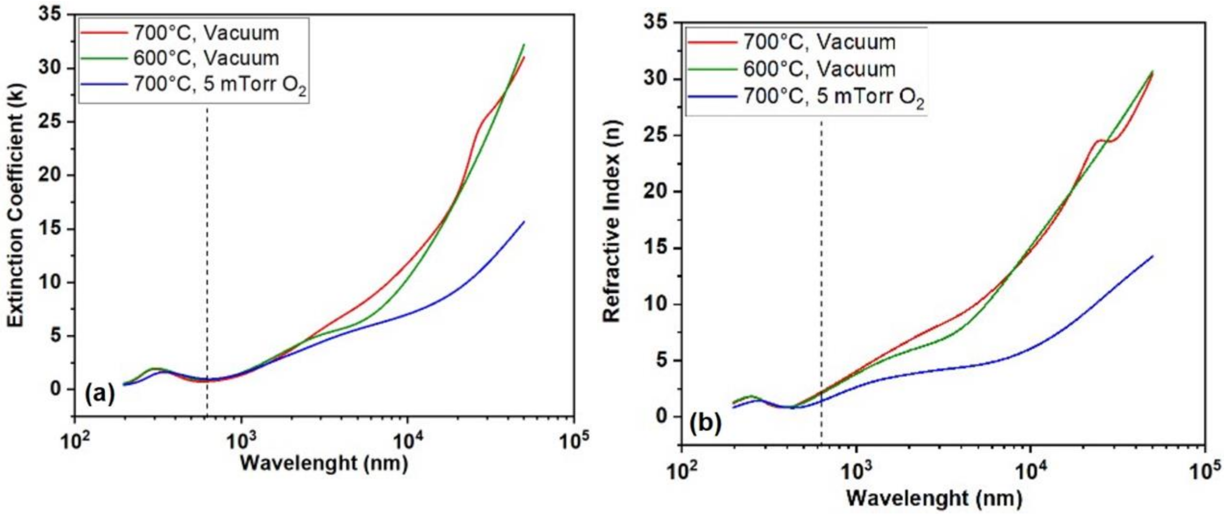

Figure S9. (a) Extinction Coefficient (k) and (b) refractive index (n) TiN/TiNO thin film samples deposited at 700°C- Vacuum, 600°C- Vacuum, and 700°C- 5 mTorr O_2.

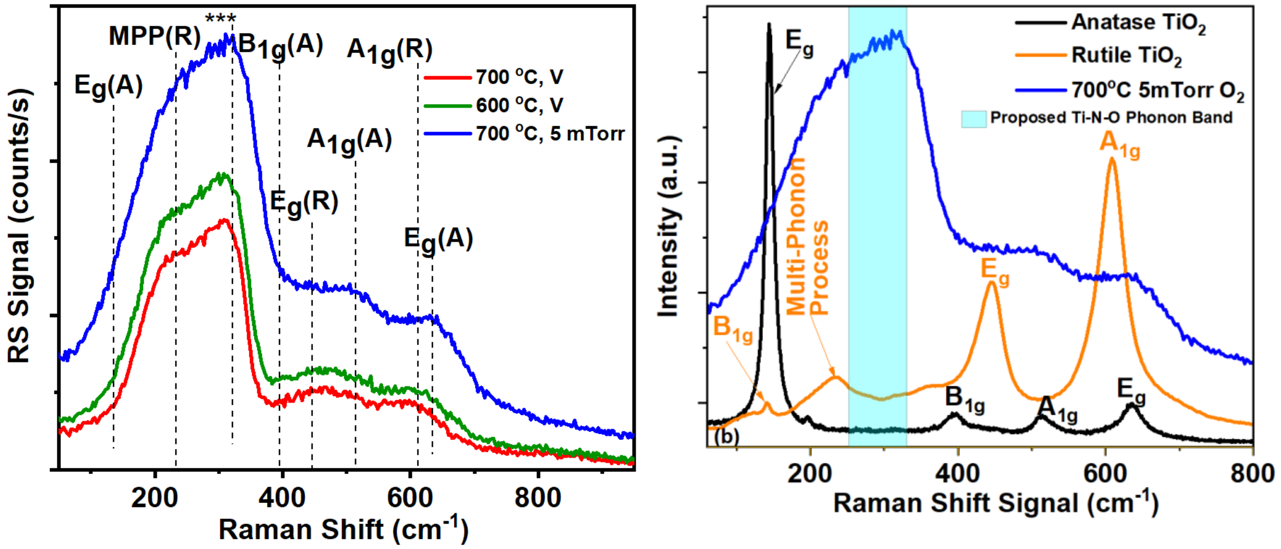

Figure S10. (a) Raman Spectra from the TiNO deposited at 700°C and 600°C in vacuum and 5 mTorr O_2 recorded using 532 nm wavelength laser excitation. E_g (144 cm^{-1}-A, 198 cm $^{-1}$-A, 446 cm^{-1}-R, 632 cm^{-1}-A), B_{1g} (140 cm^{-1}-R, 394 cm^{-1}-A), A_{1g} (514 cm^{-1}-R, 610 cm^{-1}-A), and Multi-Photon Phase-MPP (240cm^{-1}-R) and (b) Comparison of Raman Spectra recorded from PLD grown rutile and anatase TiO_2 with the TiNO films showing the proposed Ti-N-O phonon band; the 700°C- 5mTorr spectrum is magnified by ~10 units to improve visibility of spectrum.

Supporting Note S2. First-principles calculation and lattice dynamics

In this study, ab-initio calculations based on density functional theory (DFT) [5] using the Vienna Ab-initio Simulation Package (VASP) [6] were carried out to optimize the structures of anatase and rutile TiO_2 crystals. The projector-augmented wave (PAW) [7]method was employed to treat the Ti($4d^{10}5s^1$) and O($2s^2 2p^4$) shells as valence states. The Perdew-Burke-Ernzerhof (PBE) functional[8], within the framework of the generalized gradient approximation (GGA) [9], was used for the exchange-correlation functional. The ionic positions and unit cell geometry were fully optimized using a plane-wave cutoff energy of 600 eV and a 12 × 12 × 16 Monkhorst-Pack electronic k-point mesh, with a strict force convergence criterion of 10^{-5} eV·Å^{-1} and an energy convergence criterion of 10^{-8} eV. The resulting relaxed lattice constants are a = b = 4.66 Å, c = 2.97 Å for rutile, and a = b = c = 5.56 Å for anatase TiO_2 crystals, respectively. To account for long-range dipole-dipole interactions in the crystals, the dielectric tensor (ε) and the Born effective charges (Z) of both rutile and anatase TiO_2 were calculated using density functional perturbation theory (DFPT)[10]. The harmonic interatomic force constants (IFCs) were calculated using the finite-displacement approach [11] with a 3 × 3 × 3 supercell for anatase TiO_2 and a 3 × 3 × 4 supercell for rutile TiO_2, along with a 4 × 4 × 4 Monkhorst-Pack electronic k-point mesh and a plane-wave cutoff energy of 600 eV in VASP. The virtual crystal approximation (VCA) approach [12, 13]was used to model the bulk TiNO compound for lattice dynamics. In this work, the phonon calculations were performed using the Phonopy package[14].

References

(1) Söğüt, Ö.; Büyükkasap, E.; Ertuğrul, M.; Küçükönder, A. Chemical effect on enhancement of Coster–Kronig transition of L3 X-rays. *Journal of Quantitative Spectroscopy and Radiative Transfer* **2002**, *74* (3), 395-400.
(2) Ohno, M. Effects of Coster–Kronig fluctuation and decay on X-ray photoelectron spectroscopy spectra. *Journal of electron spectroscopy and related phenomena* **2003**, *131*, 3-28.
(3) Nyholm, R.; Martensson, N.; Lebugle, A.; Axelsson, U. Auger and Coster-Kronig broadening effects in the 2p and 3p photoelectron spectra from the metals 22Ti-30Zn. *Journal of Physics F: Metal Physics* **1981**, *11* (8), 1727.
(4) Bambynek, W.; Crasemann, B.; Fink, R.; Freund, H.-U.; Mark, H.; Swift, C.; Price, R.; Rao, P. V. X-ray fluorescence yields, Auger, and Coster-Kronig transition probabilities. *Reviews of modern physics* **1972**, *44* (4), 716.
(5) Hohenberg, P.; Kohn, W. Inhomogeneous electron gas. *Physical review* **1964**, *136* (3B), B864.
(6) Kresse, G.; Furthmüller, J. Efficient iterative schemes for ab initio total-energy calculations using a plane-wave basis set. *Physical review B* **1996**, *54* (16), 11169.
(7) Blöchl, P. E. Projector augmented-wave method. *Physical review B* **1994**, *50* (24), 17953.
(8) Perdew, J. P.; Burke, K.; Ernzerhof, M. Generalized gradient approximation made simple. *Physical review letters* **1996**, *77* (18), 3865.
(9) Perdew, J. P.; Burke, K.; Wang, Y. Generalized gradient approximation for the exchange-correlation hole of a many-electron system. *Physical review B* **1996**, *54* (23), 16533.
(10) Baroni, S.; De Gironcoli, S.; Dal Corso, A.; Giannozzi, P. Phonons and related crystal properties from density-functional perturbation theory. *Reviews of modern Physics* **2001**, *73* (2), 515.
(11) Esfarjani, K.; Stokes, H. T. Method to extract anharmonic force constants from first principles calculations. *Physical Review B—Condensed Matter and Materials Physics* **2008**, *77* (14), 144112.
(12) Yao, F.; Xia, S.; Wei, H.; Zheng, J.; Yuan, Z.; Wang, Y.; Huang, B.; Li, D.; Lu, H.; Xu, D. Experimental evidence of superdiffusive thermal transport in Si0. 4Ge0. 6 thin films. *Nano Letters* **2022**, *22* (17), 6888-6894.
(13) Li, W.; Lindsay, L.; Broido, D. A.; Stewart, D. A.; Mingo, N. Thermal conductivity of bulk and nanowire Mg 2 Si x Sn 1− x alloys from first principles. *Physical Review B—Condensed Matter and Materials Physics* **2012**, *86* (17), 174307.
(14) Togo, A.; Tanaka, I. First principles phonon calculations in materials science. *Scripta Materialia* **2015**, *108*, 1-5.
